# Simulation and Mechanistic Investigation of the Arrhythmogenic Role of the Late Sodium Current in Human Heart Failure

**DOI:** 10.1371/journal.pone.0032659

**Published:** 2012-03-12

**Authors:** Beatriz Trenor, Karen Cardona, Juan F. Gomez, Sridharan Rajamani, Jose M. Ferrero, Luiz Belardinelli, Javier Saiz

**Affiliations:** 1 Instituto de Investigación Interuniversitario en Bioingeniería y Tecnología Orientada al Ser Humano, Universitat Politècnica de València, Valencia, Spain; 2 Department of Biology, Gilead Sciences, Palo Alto, California, United States of America; Brigham & Women's Hospital - Harvard Medical School, United States of America

## Abstract

Heart failure constitutes a major public health problem worldwide. The electrophysiological remodeling of failing hearts sets the stage for malignant arrhythmias, in which the role of the late Na^+^ current (I_NaL_) is relevant and is currently under investigation. In this study we examined the role of I_NaL_ in the electrophysiological phenotype of ventricular myocytes, and its proarrhythmic effects in the failing heart. A model for cellular heart failure was proposed using a modified version of Grandi et al. model for human ventricular action potential that incorporates the formulation of I_NaL_. A sensitivity analysis of the model was performed and simulations of the pathological electrical activity of the cell were conducted. The proposed model for the human I_NaL_ and the electrophysiological remodeling of myocytes from failing hearts accurately reproduce experimental observations. The sensitivity analysis of the modulation of electrophysiological parameters of myocytes from failing hearts due to ion channels remodeling, revealed a role for I_NaL_ in the prolongation of action potential duration (APD), triangulation of the shape of the AP, and changes in Ca^2+^ transient. A mechanistic investigation of intracellular Na^+^ accumulation and APD shortening with increasing frequency of stimulation of failing myocytes revealed a role for the Na^+^/K^+^ pump, the Na^+^/Ca^2+^ exchanger and I_NaL_. The results of the simulations also showed that in failing myocytes, the enhancement of I_NaL_ increased the reverse rate-dependent APD prolongation and the probability of initiating early afterdepolarizations. The electrophysiological remodeling of failing hearts and especially the enhancement of the I_NaL_ prolong APD and alter Ca^2+^ transient facilitating the development of early afterdepolarizations. An enhanced I_NaL_ appears to be an important contributor to the electrophysiological phenotype and to the dysregulation of [Ca^2+^]_i_ homeostasis of failing myocytes.

## Introduction

Over 5 million persons in the United States suffer from heart failure (HF) and more than 250,000 die annually [1]. Patients with congestive HF are prone to develop complex ventricular tachyarrhythmias and some die suddenly [2]. Experimental studies conducted using animal models of HF have shown that ventricular arrhythmias are mainly due to non-reentrant mechanisms, most likely triggered activity based on afterdepolarizations [2].

Much attention has been paid to the understanding of the arrhythmogenic mechanisms induced by the structural, electrical, and metabolic remodeling of the failing heart. The electrophysiological remodeling of the failing heart has been well described (see [3], [4] for review). Action potential (AP) prolongation, altered Ca^2+^ handling, as well as intracellular Na^+^ ([Na^+^]_i_) accumulation have been established as the hallmark characteristics of myocytes and tissues isolated from failing human and canine hearts [5]–[8]. These alterations are closely related to arrhythmogenic mechanisms, such as early (EADs) and delayed (DADs) afterdepolarizations, observed in HF [9]. Functional remodeling of ion channels and pumps is the underlying cause for AP duration (APD) prolongation and altered intracellular Ca^2+^ ([Ca^2+^]_i_) homeostasis. Downregulation of outward K^+^ currents is the most consistent change in animal models and human HF [3], [4], [6], [7], [10]. Major changes in intracellular and sarcoplasmic reticulum (SR) Ca^2+^ homeostasis are also associated with HF in several animal species, included human [9], [11]–[13]. In myocytes from failing hearts [Na^+^]_i_ concentration and Ca^2+^ handling are closely linked; [Na^+^]_i_ is increased in failing ventricular myocytes from human and other animal species [3], [4], [9], [14] and a prominent increase of the human late Na^+^ current (I_NaL_) has also been documented [15], [16], and has been proposed as a therapeutic target [17]–[19]. Experimental studies have shown that the I_NaL_ is involved in the generation of EADs in myocytes [17], [18] and life-threatening arrhythmias, such as torsade de pointes (TdP) [20], especially under conditions of reduced repolarization reserve in several animal species [21]–[23]. Other changes in biomarkers for arrhythmic risk such as the increase in the reverse rate-dependent APD prolongation [20], [23], [24] have been attributed to an increase in I_NaL_ concomitant with inhibition of outward K^+^ currents.

Thus, the goal of our study was to analyze, using computer simulations, the role of the I_NaL_ in the setting of human HF. A mathematical model of human HF is proposed at cellular level using a modified version of the Grandi et al. model (from herein referred to as the GPB model) [25] of endocardial AP, in which a new formulation of the I_NaL_ is included. The sensitivity analysis performed for the HF model, as well as the simulations of the rate-dependence of APD and EAD generation revealed that the mechanisms underlying the arrhythmogenic processes taking place in HF conditions, from a theoretical point of view, include an enhanced I_NaL_.

## Results

### Effects of I_NaL_ on human AP

**Figure 1:**
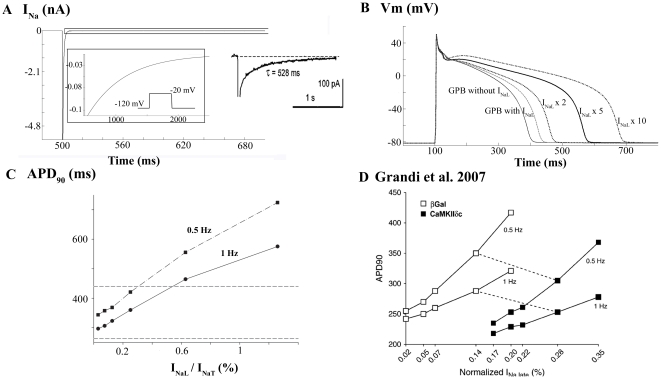
Time course of late Na^+^ current and its effects on AP. A: Simulation of late Na^+^ current (I_NaL_) using a voltage clamp protocol similar to that of the experimental measurements obtained by Maltsev et al. [26] (shown in the right inset) in ventricular myocytes at room temperature. The left inset shows an expanded view of the current between 480 ms and 2500 ms. B: Simulated action potentials (APs) at 1-Hz pacing rate using the GPB model, the GPB model modified with control I_NaL_, and I_NaL_ enhanced 2-fold, 5-fold and 10-fold. C: APD_90_ sensitivity to the I_NaL_ amplitude. APs were simulated at 0.5-Hz (square symbols) and 1-Hz (circle symbols) pacing rate by varying I_NaL_/I_NaT_ ratio from 0.0298% to 1.26%. The range of experimental APD at 90% repolarization (APD_90_) for human is represented by the two discontinuous lines. D: APD_90_ sensitivity to the I_NaL_ amplitude (open symbols) taken from Grandi et al. [30] who used a rabbit model.

Figure 1A shows the generation of I_NaL_ trace using a voltage clamp protocol similar to that of Maltsev et al. [26] in ventricular mid-myocardial myocytes at room temperature. The simulated I_NaL_ had similar time evolution and amplitude to the experimental findings. Panels B and C show the effects of this current on AP and APD at 90% of repolarization (APD_90_), respectively. The inclusion of I_NaL_ in the GPB model slightly prolongs steady-state APD at 1 Hz (see Figure 1B). APD remains within the physiological range for human endocardial myocytes [8], [27], [28]. By increasing the magnitude of I_NaL_ by a factor of two-, five- or ten-fold, we obtained APD_90_ prolongations of 11%, 44% and 78%, respectively. Experimental recordings of monophasic APs (MAPs) under the effects of veratridine, an enhancer of I_NaL_, show similar effects on APD prolongation in rabbit myocytes [29]. We determined the sensitivity of APD_90_ to I_NaL_ amplitude at different stimulation rates. As shown in Figure 1C, I_NaL_/I_NaT_ ratios, where I_NaT_ is the transient peak I_Na_, were varied from 0.0298% to 1.26% and APD_90_ significantly increased according to the magnitude of I_NaL_. Furthermore, the sensitivity of APD_90_ increased with the slowing of the stimulation rate (compare 1 Hz and 0.5 Hz). Similar simulations were carried out in a rabbit model of AP [30]. As shown in Figure 1D, the difference in sensitivity of APD_90_ to changes in frequency of stimulation (0.5 and 1 Hz) was also observed. Note that the morphology of the curves in Figure 1D is different to our results, as different models for I_NaL_ and different AP models were used. The ratio chosen in our model of 0.12% yields an APD_90_ that is within the physiological range for 1 Hz indicated by the discontinuous lines.

### Heart Failure model and sensitivity analysis

**Figure 2:**
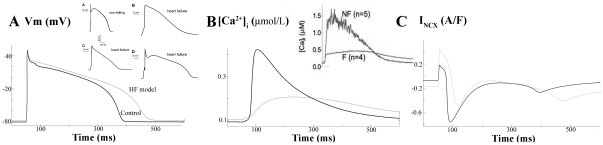
Electrophysiological changes in heart failure. Simulated APs (panel A), [Ca^2+^]_i_ transients (panel B), and Na^+^/Ca^2+^ exchanger (NCX) activity (panel C) at 1-Hz pacing rate in control (dark line) and in heart failure (HF) conditions (light line). The insets show experimental recordings of Priebe and Beuckelmann et al. [5] (panel A) and Weber et al. [31] (panel B).

APs and [Ca^2+^]_i_ transients were simulated under conditions of HF. Figure 2 shows APs (panel A), [Ca^2+^]_i_ transients (panel B) and Na^+^/Ca^2+^ exchanger (NCX) function (I_NCX_) (panel C) of failing (light line) and nonfailing (dark line) ventricular myocytes after achieving steady-state conditions for a stimulation rate of 1 Hz. Our results showed an APD_90_ prolongation of 24% in failing myocytes versus normal ones, as well as a 18% prolongation in APD_50_, so that triangulation (APD_90_–APD_50_) increased by 43% under HF conditions. The experimental observations shown in the inset of Figure 2 (panel A) taken from reference [5], show the variability of the APD in failing myocytes from human hearts, which falls within the simulated values of APD observed.

Figure 2B depicts an altered [Ca^2+^]_i_ transient under HF conditions, as has been documented experimentally and is shown in the inset [31]. Indeed, diastolic [Ca^2+^]_i_ is slightly increased, whereas peak systolic [Ca^2+^]_i_ is reduced to 41% of the one observed during normal conditions. An additional reported feature of the [Ca^2+^]_i_ transient of a failing myocyte is its slow decay. In our simulations Ca^2+^ transient decay, quantified as the time needed from the peak value to reach 10% of the transient amplitude (ô_Ca_ decay), yielded 630 ms and 380 ms in the failing and nonfailing myocytes, respectively. Finally, Figure 2C illustrates the changes in I_NCX_ during HF, mainly a shift in the time of the reversal potential for the NCX (t_NCXRP_) of 20 ms. Similar shifts in t_NCXRP_ have been reported in experimental studies [31].

**Figure 3:**
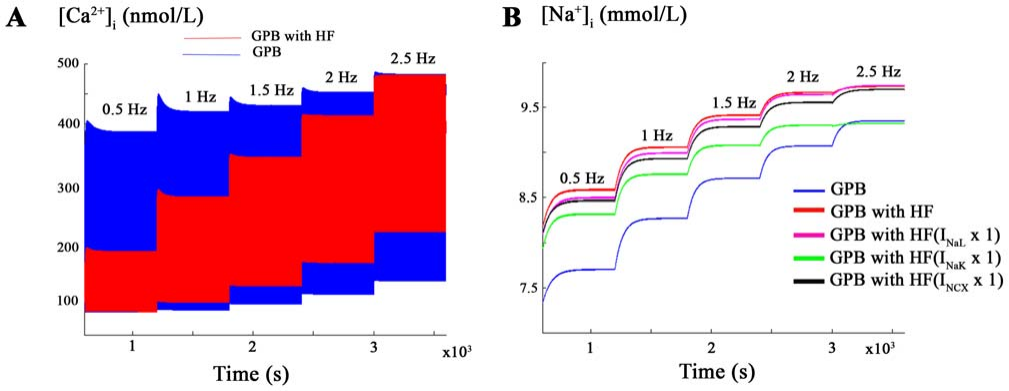
[Ca^2+^]_i_ and [Na^+^]_i_ changes with increasing frequency in HF. Influence of the stimulation rate on [Ca^+2^]_i_ (panel A) and [Na^+^]_i_ in nmol/L (panel B) determined using the staircase protocol. The simulations were performed using the modified GPB model with I_NaL_ incorporated in normal conditions (blue) and the modified GPB model incorporating HF conditions (red). In panel B, conditions of HF without I_NaL_, I_NaK_ or I_NCX_ remodeling are also shown.

Changes in [Na^+^]_i_ and [Ca^2+^]_i_ levels under HF conditions at various stimulation rates were also investigated using a staircase protocol described in the methods section and described previously [32]–[34]. Figure 3A shows that diastolic [Ca^2+^]_i_ level is higher in HF than in normal conditions and systolic level is always higher in normal conditions than in HF, as reported experimentally [5], [35], [36]. The impact of the variability of ion channel remodeling on these results are presented in Figures S4, S5, S6, S7.

Another feature of myocytes from failing hearts is the high concentration of [Na^+^]_i_ regardless of the stimulation rate, as observed in Figure 3B and experimentally reported by Pieske et al. [33] in failing human myocardial cells. However, as acknowledged by Pieske et al. [33], it should be noted that the absolute [Na^+^]_i_ values in their experimental work were overestimated in normal and failing hearts. The impact of the variability of ion channel remodeling on these results are presented in Figures S8 and S9.

To analyze the mechanisms responsible for [Na^+^]_i_ accumulation with frequency in failing myocytes, the above pacing protocol was applied for a) normal conditions (blue), b) HF conditions (red), c) HF with no I_NaL_ upregulation (purple), d) HF with no Na^+^/K^+^ pump current (I_NaK_) downregulation (green) and e) HF with no I_NCX_ upregulation (black). From the results obtained in Figure 3B, it can be deduced that [Na^+^]_i_ accumulation is mainly driven in HF by alterations in I_NaK_, and much less by the I_NCX_ and I_NaL_.

**Figure 4:**
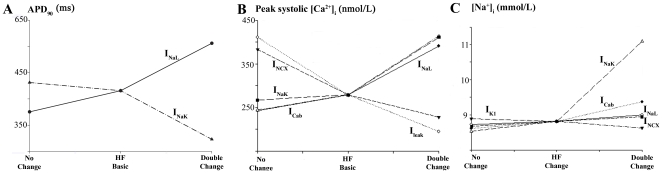
Sensitivity of electrophysiological parameters to changes in ionic current properties. Changes in APD_90_ (panel A), peak systolic [Ca^2+^]_i_ (panel B), and [Na^+^]_i_ (panel C) with changes in I_NaL_, I_NaK_, I_leak_, I_NCX_, I_K1_ and I_Cab_, as labeled next to each curve. Axis x represents the simulation conditions; for “HF Conditions” the remodeling of the basic HF model is considered, for “No Change” the labeled current is unchanged as it is in the GPB model, for “Double Change” the labeled current undergoes a double change with respect to the change exerted in “HF conditions.”

**Figure 5:**
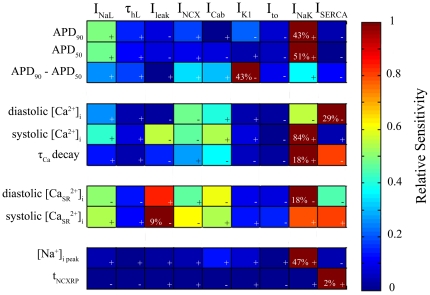
Relative sensitivities of the electrophysiological parameters to changes in ionic current properties. Dark blue color indicates lack of dependency between the ionic property (1^st^ row) and the EP parameter (1^st^ column), and dark red color indicates strong direct (+) dependency or inverse dependency (−). Percents in each box indicate the maximum absolute sensitivity of the EP parameter correspondent to that row for all ionic properties.

Our basic HF model, as described in Figure 2 and 3, reproduced the main changes in the electrophysiological (EP) characteristics of myocytes from failing hearts. However, the experimentally reported ranges for EP changes, as well as the magnitude of ion channels, transporters and respective current remodeling, vary substantially for different experimental settings and HF stages. Hence, we performed a sensitivity analysis to assess the impact of the main ionic parameters remodeled in HF on the described EP characteristics. Figure 4A highlights the role of I_NaL_ and I_NaK_ in APD_90_ under conditions of HF. When I_NaL_ undergoes a 2-fold increase (i.e. doubling) with respect to the basic HF increase, APD is prolonged 22% and no change at all in this current leads to a shortening of the APD_90_ by 10% with respect to basic HF conditions. This result is in agreement with experimental recordings in failing human myocytes, where I_NaL_ has a crucial role in APD [19]. Similarly, the downregulation of Na^+^/K^+^ pump in HF has a relevant effect on APD_90_ shortening, and further reduction of the current leads to a decrease of 22% with respect to HF APD_90_ value, which is in agreement with experimental observations [37]. Figure 4B reveals the importance of I_NaK_, I_NCX_, the background Ca^2+^ current (I_Cab_), the leak Ca^2+^ current (I_leak_), and I_NaL_ in determining the value of peak systolic [Ca^2+^]_i_ during HF. Regarding the regulation of [Na^+^]_i_ value in failing myocytes, Figure 4C shows the important role of I_NaK_, I_Cab_, the inward rectifier K^+^ current (I_K1_), I_NCX_ and I_NaL_. To summarize the sensitivity of some of the EP characteristics (1^st^ column) during HF to the altered ionic parameters (1^st^ row), Figure 5 shows the relative sensitivity normalized to the maximum sensitivity for that particular characteristic, as described in the methods section. The positive and negative signs indicate whether the change of the ionic current and the HF EP characteristic follow the same or inverse tendency, respectively. Percentages in each box indicate the maximum absolute sensitivity of the EP parameter correspondent to that row for all ionic properties. From this sensitivity analysis it could be deduced that APD was particularly sensitive to I_NaL_ and to I_NaK_ (green and burgundy colors in rows 1 and 2). Furthermore, I_K1_, I_NCX_ and I_NaK_ have an important effect on AP triangulation. The main features of Ca^2+^ transient in HF (3 medium rows) were mainly influenced in this order by I_NaK_, SERCA function, I_NCX_, I_leak_, I_Cab_, and I_NaL_. The SR Ca^2+^ concentration ([Ca^2+^]_SR_) is also influenced by other currents but in this case I_leak_ becomes more important than I_NCX_. [Na^+^]_i_ is mainly regulated by I_NaK_, I_Cab_, I_K1_, I_NCX_ and I_NaL_. Finally, t_NCXRP_ is mainly modulated by the SR Ca^2+^-ATPase activity (I_SERCA_). The absolute effects of small changes (±15%) in the ionic remodeling of the basic HF model on the main results of our simulations are shown in Figures S1, S2, S3, S4, S5, S6, S7, S8, S9, S10, and S11. These absolute effects are in agreement with the relative role of the ionic parameters described above. We have especially focused on the sensitivity of APD_90_ in failing myocytes to I_NaL_/I_NaT_ ratio, as shown in Figure S1. In this figure, the values of I_NaL_/I_NaT_ ratios for 1 Hz in the modified the GPB model and in our basic HF model are indicated by arrows and are within experimental ranges (indicated by blue dotted lines in normal myocytes and by red discontinuous lines in failing myocytes, respectively). Changes in the selected ratios affect the APD_90_ values of failing and normal myocytes, which stay within experimental ranges provided that the changes of these ratios are not very large.

### APD rate-dependence and the role of I_NaL_ in HF

**Figure 6:**
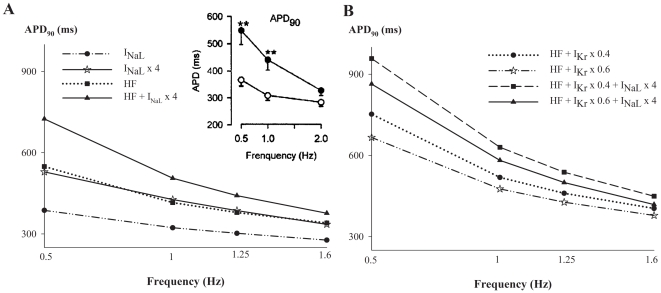
Role of I_NaL_ in APD rate-dependence in HF and reduced repolarization reserve. Simulated APD dependence on stimulation frequency for normal (circles in panel A) and HF (squares in panel A) conditions and in the presence of enhanced I_NaL_ in normal (stars in panel A) and HF (triangles in panel A) conditions. The inset shows experimental results of Li et al. [7]. Panel B, depicts the APD rate-dependence for HF combined with different degrees of I_Kr_ inhibition and I_NaL_ enhancement.

The role of I_NaL_ in steady-state APD_90_ rate-dependence during HF was analyzed. An enhanced reverse frequency-dependence of APD_90_ is considered to be a major proarrhythmic risk (see [38] for review). We performed simulations and measured APD_90_ under normal and HF conditions for different magnitudes of I_NaL_ activation (see Figure 6A). Also 40% and 60% inhibition of the rapid component of the delayed rectifier K^+^ current (I_Kr_) in failing myocytes were simulated with different degrees of I_NaL_ enhancement (see Figure 6B). Indeed, the concomitant alteration of I_NaL_ and I_Kr_ has been reported to have important effects on arrhythmogenicity [20]–[24]. An important result obtained in our simulations, as shown in Figure 6A, is the reverse rate-dependence effect on APD_90_ exerted by I_NaL_ enhancement for normal conditions (compare circles with stars). The rate-dependence yielded 110 and 190 ms maximal APD_90_ prolongation in normal conditions versus I_NaL_ increased by 4-fold, respectively. Similar observations were reported by others [20], [24] in rabbit ventricular myocytes under the effects of I_NaL_ activators, as well as in isolated perfused rabbit heart [22]. When considering HF conditions (squares), the rate-dependence is also increased with respect to normal conditions. The ΔAPD value is very similar to the value obtained with I_NaL_ enhanced (stars). This implies that I_NaL_ might be the main driver of the increased reverse rate-dependent prolongation of APD. Additionally, greater magnitudes of I_NaL_ (black triangles) are associated with greater increase in the reverse rate-dependency of APD of failing myocytes. Experimental studies [7] have also documented the increase of APD rate-dependence in human failing epicardial myocytes, as depicted in the inset. Simulations of APD rate-dependence in HF caused by small changes (±15%) in the ionic remodeling of the basic HF model (see Figures S2, S3, S4, S5, S6, S7, S8, S9, S10, and S11) to take into account the experimental variability on electrical remodeling during HF were also performed. The results do not change significantly with respect to the basic HF model, except for I_NaK_ and I_NaL_, which have an important effect on APD_90_, as predicted by the sensitivity analysis shown in Figure 5.

Figure 6B summarizes the concomitant contribution of I_NaL_ enhancement and I_Kr_ inhibition in the reverse rate-dependency of APD, shown in various experimental studies [20], [24], in failing myocytes.

**Figure 7:**
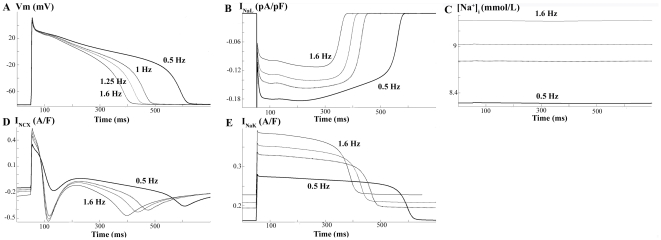
Mechanisms for APD rate-dependence in HF. Simulated APs and ionic currents at different stimulating frequencies (0.5, 1, 1.25 and 1.6 Hz) under HF conditions.

To analyze the ionic mechanisms responsible for steady-state APD rate-dependence in failing myocytes, we determined the behavior of several ionic currents (I_NaL_, I_NCX_ and I_NaK_) and [Na^+^]_i_ at different frequencies of stimulation, as depicted in Figure 7. Our results show that the shortening of the APD with increased frequency in HF is explained on the one hand by the decrease in I_NaL_, indeed faster rates lead to an incomplete recovery from inactivation of this current [39]–[41], and on the other hand by [Na^+^]_i_ accumulation leading to an increase of I_NaK_. The role of I_NCX_ on APD is not evident as both outward and inward modes are enhanced. Although several experimental studies suggest the direct involvement of this current on APD shortening with frequency, as will be discussed later, its implication in our simulations is not evidenced. Indeed, when we stimulated the cell at high frequency (1.6 Hz) but clamped [Na^+^]_i_ value to the lower [Na^+^]_i_ value corresponding to the low frequency (0.5 Hz), we observed a decrease in APD_90_ with respect to APD_90_ at low frequency, concomitant with a decrease and increase of the outward and inward modes of NCX activity, respectively (results not shown). These changes in I_NCX_ should lead to a longer APD_90_, instead of shorter, suggesting that I_NCX_ is not determinant in APD rate-dependence in the present model.

### Arrhythmogenic effects of I_NaL_

**Figure 8:**
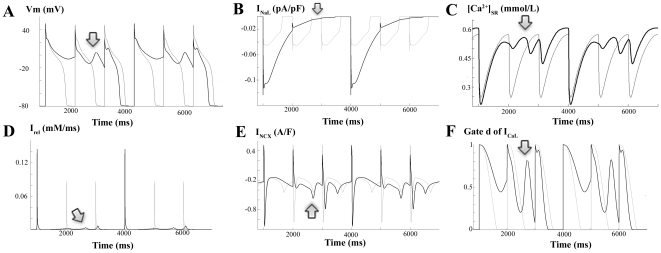
Mechanisms for early afterdepolarizations with enhanced I_NaL_. Simulated APs and ionic currents at a stimulating rate of 1 Hz for HF conditions, 50% inhibition of I_Kr_ and 30% increase of I_CaL_. Panel A shows early afterdepolarizations (EADs; dark line) when I_NaL_ was doubled and APs with no EADs when I_NaL_ was normal (light line). The temporal evolutions of I_NaL_ (panel B), I_rel_ (panel D), I_CaL_ (panel C), NCX activity (panel E), and activation gate of I_CaL_ are also depicted when I_NaL_ was doubled (dark line) and when I_NaL_ was normal (light line).

Under basic HF conditions, APD prolongation favors the occurrence of EADs. We were therefore interested in determining the role of I_NaL_ in the repolarization abnormalities of human failing hearts. We stimulated the myocyte at 1 Hz and simulated conditions of low repolarization reserve prone to EADs generation. Under HF conditions using the basic HF model, I_Kr_ was reduced by 50% and the Ca^2+^ current (I_CaL_) was increased by 30%. Figure 8A shows EADs (dark line) when I_NaL_ was doubled, whereas APs displayed no EADs when I_NaL_ was normal (light line). The impact of the variability in ion channel remodeling on EADs generation can be observed in Figures S10 and S11. The mechanisms by which EADs arise are dictated by a very delicate balance of ionic currents during AP plateau. Slight changes in this balance can suppress EADs, as occurs in the case when all ionic parameters are reduced in 15% with respect to the basic HF model (see Figure S11).

Figure 8 depicts APs and ionic currents to provide insight into the mechanisms by which enhancement of I_NaL_ indirectly triggers EADs [17]. The APD prolongation (panel A) caused by enhancement of I_NaL_ (panel B) leads to the reactivation of the activation gate d (panel F) of the I_CaL_, which triggers the EAD, as was suggested by January et al. [42]. However, the important contribution of the altered Ca^2+^ transient should also be taken into consideration. Indeed, the APD prolongation caused by I_NaL_ enhancement alters Ca^2+^ handling. As observed in panel C, [Ca^2+^]_SR_ remains elevated and provokes the trigger of a small spontaneous release (panel D, arrow) just before the EAD arises (panel A, arrow). NCX (panel E) extrudes Ca^2+^ operating in the inward mode, which contributes to membrane potential increase. This would contribute to I_CaL_ reactivation which triggers the EAD. The involvement of these currents is also shown in Figures S12, S13, S14, and S15, as a partial block of SR Ca^2+^ release (I_rel_) or I_NCX_ suppresses the EADs.

## Discussion

### Major Findings

In this article, one of the most recent and detailed AP model for human ventricular myocytes, the GPB model, was modified to simulate HF functional remodeling. The I_NaL_ was formulated and introduced in the model on the basis of human ventricular voltage-clamp data. An important novelty of the study is the sensitivity analysis of the modulation of the main EP characteristics due to ion channel and transporters remodeling, highlighting the role of I_NaL_ in the changes of APD, triangulation and Ca^2+^ transient under HF conditions. A mechanistic investigation of [Na^+^]_i_ accumulation and APD shortening with increasing stimulation frequency in failing myocytes, revealed the roles of I_NaK_, I_NCX_ and I_NaL_. Finally, the arrhythmogenic effects of increased I_NaL_ were investigated. Our results showed the important role of I_NaL_ in APD reverse rate-dependence and EAD generation in failing myocytes, representing the first realistic simulation study on this issue, which complements experimental studies and sheds light into the ionic mechanisms responsible for HF phenotype.

### Simulation of heart failure

Recently, O'Hara et al. [43] published a new AP model for human ventricular myocytes; however, in the present study the GPB model was chosen because its behavior was adequate to analyze the changes exerted in HF in APD, Ca^2+^ transient, the APD rate-dependence and EADs generation. Nevertheless future studies comparing the behavior of both models in pathological situations should be conducted. The GPB model was developed using human voltage-clamp data, and validated against recent experimental results [25]. In the present study, we demonstrate for the first time that the GPB model, subject to ion channel and transporter remodeling, is also appropriate to reproduce the electrical behavior of failing myocytes.

A large body of experimental research has focused on the functional remodeling of failing hearts [3], [4], [6], [7], [10], [11], [14]. However, little has been done in this field from a theoretical perspective. One of the first human ventricular AP models, including HF remodeling, was formulated by Priebe and Beuckelmann in 1998 [5]. However, I_NaL_ was not considered, and the formulation of Ca^2+^ handling was not as realistic as it is in the GPB model. This represents an important limitation for HF, when Ca^2+^ transient undergoes critical changes. Later, in 2005, Zhang et al. [44] modified the human ten Tusscher model [45] to simulate HF. In the latter model, I_NaL_ was considered, but with experimental data derived using dog ventricular myocytes [46], and remodeling was derived using experimental data from multiple species. However, controversy about the changes introduced in their model for I_NaT_, the slow component of the delayed rectifier K^+^ current (I_Ks_) or I_CaL_ exists for human failing myocytes [2]–[4]. Other authors have simulated ion remodeling of myocytes from canine and rabbit failing hearts [12], [47] that reproduce the APD and Ca^2+^ transient changes associated with HF. Our model of basic HF not only reproduces experimental observations on APD prolongation and Ca^2+^ transients alterations [5], [7], [11], [31], but also the APD, [Ca^2+^]_i_ and [Na^+^]_i_ rate-dependence reported in failing myocytes [33], [35], [36]. The ionic model of ventricular HF presented in this study is not only based on recent human experimental data, but also takes into account the variability documented in EP studies using sensitivity analysis. Sensitivity analysis also elucidates the ionic mechanisms responsible for the main changes in the EP characteristics of HF. Previous sensitivity analysis have been performed using the human ten Tusscher et al. [48] AP model [32], a modified version of the GPB model [34] and O'Hara et al. model [43], as well as rabbit [48] AP model. In these studies, the authors analyzed the effects of the physiological variability of the main ionic properties on several electrophysiological characteristics and biomarkers for arrhythmic risk, under normal physiological conditions. The main similarities of our results in HF with their studies on normal myocytes, are the importance of I_NaK_ [32], [34] and I_NaL_ [43] in regulating APD_90_, and I_K1_ [32], [34] and I_NaK_ in regulating AP triangulation. The variability of I_CaL_ and I_Kr_ has also an important effect in their studies but was not considered in HF, as these currents are not remodeled. Also the impact of I_NaK_ and I_NCX_ was important on Ca^2+^ transients under normal conditions [32], [34].

Regarding the variability of functional remodeling in HF, it is worth noting that experimental measurements reported in literature have been conducted in different stages of HF (see [3], [4] for review). In the present study, ionic currents are within experimental ranges, but do not take into account differences in various stages of HF. Different combinations of changes were tested in the sensitivity analysis in order to determine the relative effects on the EP characteristics in HF (Figure 5) and the absolute effects of small changes (±15%) in ionic parameters on APD-rate-dependence (Figures S2 and S3), [Ca^2+^]_i_ transients (Figures S4, S5, S6, and S7), [Na^+^]_i_ values (Figures S8 and S9) and EADs generation (Figures S10 and S11). It should be noted that the results of the present sensitivity analysis are dependent on the values chosen for the ionic parameters in the basic HF model, as the percents of change also depend on these chosen values. The results suggest that the downregulation of the Na^+^/K^+^ pump has an important role in the electrophysiological and Ca^2+^ transient changes in HF, highlighting the need for new experimental data. Electrophysiological studies [49] conducted in failing human hearts revealed increased Ca^2+^ transients under the effects of strophanthidin, a Na^+^/K^+^ pump blocker, which is in agreement with our results. However, little is known about the effects of Na^+^/K^+^ pump activity on APD in HF. Experiments carried out on hypertrophic rat hearts by Levi et al. [37] showed that the application of ouabain, a Na^+^/K^+^ pump blocker, resulted in APD shortening, which was caused by [Na^+^]_i_ accumulation and the subsequent increase in the reverse mode of the NCX. Similar studies should be performed using human failing hearts to determine the role of the effects of Na^+^/K^+^ pump on APD. Also the [Na^+^]_i_ accumulation with increasing frequency during HF appears to be mainly due to the downregulation of this pump, and a small contribution of I_NaL_ and I_NCX_ upregulation (see Figure 3B). Experimental studies reporting [Na^+^]_i_ accumulation in human HF [33] or animal species [35], [50] suggest that I_NaK_ downregulation, I_NaL_ and I_NCX_ upregulation, and altered activity of the Na^+^/H^+^ exchanger (not included in the GPB model), might be involved. However, no experiments have been performed to in human failing myocytes to clarify the responsible mechanisms. Other simulation studies [30], [51] using the Shannon et al. rabbit AP model [52] have also reported the small and important influence of I_NaL_ and I_NaK_, respectively, on [Na^+^]_i_ accumulation with frequency under pathological situations other than HF.

Our results also reveal the role of I_NaK_ in APD rate-dependence as stated by Carmeliet [53] and by Eisner et al. [54] in their review on AP rate adaptation as well as shown in the theoretical and experimental findings by Pueyo et al. [55]. Additional simulation studies using different human AP models have also demonstrated this fact in normal conditions [32], [43]. Although O'Hara et al. [43] and Carro et al. [34] human AP models exhibit an improved behavior in the fast phase of APD rate adaptation than the GBP model, our study focused on the steady-state APD rate-dependence, where the GPB model is valid except for low frequencies [43]. Also Faber and Rudy [56] showed that APD shortening at high rates in a guinea pig AP model was due to the increase of the outward NCX activity. This observation is also consistent with experiments conducted on myocytes from human failing hearts [49], where increases in [Na^+^]_i_ led to APD shortening provoked by an increase of the reverse mode of I_NCX_. However, the GPB model exhibits a much shorter outward mode of NCX than the Faber and Rudy model. Thus its contribution to AP repolarization is not evident in the present study. Furthermore, in failing human myocytes, Ca^2+^ influx via NCX is prominent during most of the plateau phase [31]. Thus changes in I_NCX_ formulation of the GPB model should be addressed to better understand the role of this current in HF APD rate-dependence. Also new experiments in failing human myocytes demonstrating the role of I_NaK_ and I_NCX_ in APD rate-dependence would be of great interest. This finding also suggests that the functional increase of these outward currents caused by [Na^+^]_i_ accumulation may limit AP prolongation in HF, counterbalancing the increase of I_NaL_.

Our results also highlight the pivotal role of I_NaL_ in the changes of EP characteristics in HF, which will be discussed in the following section. Finally, another mechanistic effect of relative importance in HF, revealed by the simulations of the present study is the increase of AP triangulation associated with I_K1_ downregulation. Similar observations have been reported in ventricular myocytes from normal dogs when I_K1_ was reduced with barium [57] and in the sensitivity analysis performed by Romero et al. [32] and Carro et al. [34] in normal physiological conditions.

### Important role of I_NaL_ during heart failure and clinical implications

The pivotal role of I_NaL_ in the changes of EP characteristics and in arrhythmogenesis in HF has been uncovered in the present work. The formulation of the I_NaL_ model and its introduction in the GPB model was essential to draw our conclusions. Previous formulations have been proposed to model the behavior of I_NaL_ for different animal species using the Hodgkin-Huxley or Markov formalisms [19], [20], [30], [46], [58], [59]. Noble and colleagues [59] in 1998 formulated the I_NaL_ for guinea pig by adding to their AP model a Na^+^ current with reduced conductance and slow inactivation. Similarly, other Hodgkin-Huxley formulations have been proposed for rabbit [20] and dog [19], [46]. To avoid the complexity of Markov models for I_NaL_ [30], we adapted the Hodgkin-Huxley mathematical description of Hund et al. [46] to human data, as we did in a previous work using ten Tusscher et al. AP model [48]. Our model has been validated against experimental recordings of human APD in normal conditions [8], [27], [28], APD prolongation under the effect of drugs [29] and voltage clamp experiments [58]. Recently, a new AP model for human ventricular myocytes has been published by O'Hara et al. [43] including a formulation for I_NaL_ as in the present work. However, as their AP model includes CaMK regulation, their formulation of I_NaL_ also includes the effect of CaMK regulation.

In agreement with previous experimental observations, the upregulation of I_NaL_ during HF [15] or its enhancement in situations of reduced repolarization reserve [21] prolongs APD, causes AP triangulation and increase the reverse rate-dependent prolongation of APD, which are important harbingers for cardiac arrhythmias. The contribution of I_NaL_ in the reverse APD rate-dependence was also previously simulated by our group [60] using a different human AP model [48] and conditions of reduced repolarization reserve. In regard to HF conditions, there is background of decreased outward currents, and even a small increase of I_NaL_ becomes more efficient in prolonging APD and favoring the trigger of EADs. The high probability of formation of EAD, leading to TdP, during HF has been experimentally demonstrated [7] but few studies have addressed the role of I_NaL_ in such situations [18], [26], [61]. Our results are in keeping with experimental findings, in which the I_NaL_ blocker ranolazine, effectively suppresses EADs from ventricular myocytes from failing hearts [18], [23]. Furthermore, during conditions of reduced repolarization reserve, in which outward K^+^ currents are inhibited by drugs or by diseases, results of experimental studies [21]–[23], [62] have unmasked the role of endogenous or enhanced I_NaL_ in exerting proarrhythmic effects. This is in agreement with the results of our simulations, and provides evidence of this effect in HF. Our findings support the hypothesis that I_NaL_ represents an important target for triggered-arrhythmias treatment [13]. Similarly, inhibition of CaMKII, which is known to be responsible for I_CaL_ and ryanodine receptor phosphorylation as well as I_NaL_ regulation, appears to be an important therapeutic target for suppressing arrhythmias in HF [13], [17], [63].

### Limitations of the study

Several limitations need to be considered, when drawing conclusions from the present study. The model for I_NaL_ was formulated on the basis of voltage-clamp human data for mid-myocardial cells [58], as no measurements were available for endocardial or epicardial cells. Indeed, I_NaL_ is difficult to record due to the very low magnitude (∼30–60 pA) of this current under normal conditions [58]. Maltsev and Undrovinas reported a double exponential decay of I_NaL_ [58]. However, we chose a single-exponential decay formulation as proposed in previous studies [46], [64], and also in the new AP model by O'Hara et al. [43], as this model was able to reproduce its main effects on the AP. To build the model of HF used in the present study, the ionic remodeling was mainly based on experimental data observed in human hearts. Data from a large number of experimental studies have been taken into account, thus resulting in a high variability not only in the ionic remodeling but also in the stage of HF. In addition to the difficulties associated with gaining access to human hearts, explanted diseased hearts are usually in the end stage of HF. Moreover, there are controversies regarding specific ion channel currents remodeling during HF, such as I_NaT_, I_CaL_ I_Kr_, and I_Ks_, and changes in these currents were not included in our HF model. Their effects on the EP characteristics would be significant contributors to the phenotype. We did however simulate changes in I_NaL_ concomitant with inhibition of I_Kr_ and increase in I_CaL_ (Figures 6 and 8). Finally, our basic HF model has the inherited limitations described for the GPB model. In this sense, improvements related to the rapid phase of APD rate adaptation have been accomplished in later human AP models [34], [43]. The response of failing myocytes to abrupt changes of the stimulation rate was however out of the scope of the present study because of the lack of availability of experimental data in HF. With regard to steady-state APD rate-dependence, the GPB model is accurate except for very low frequencies [43]. Thus the results obtained for APD_90_ at low frequencies should be taken with caution.

### Conclusion

This study aimed to investigate in silico the role of I_NaL_ in the electrophysiological and Ca^2+^ homeostasis phenotype of myocytes from failing hearts. Our results showed that the enhancement of this current during HF can lead to important prolongation of APD and triangulation, increases in reverse rate-dependent prolongation of APD, significantly contributes to Ca^2+^ handling changes and has an indirect but pivotal role in the genesis of EADs.

## Methods

### Model of the human ventricular I_NaL_

To simulate the electrical activity of human ventricular myocytes, the AP model formulated by Grandi et al. [25] for endocardial cells was used. This model is one of the latest and most detailed mathematical model for ionic currents and Ca^2+^ handling of the human ventricular AP. A particular strength of the GPB model is its ability to reproduce the rate-dependence of APD upon outward K^+^ currents block and their individual role in repolarization. Thus, this model provides a powerful tool to explore repolarization abnormalities under conditions of disease, such as HF. However, in order to realistically simulate HF, an important issue remains unresolved in this model, namely the role of I_NaL_.

We included in the GPB model a new formulation for human I_NaL_ that is described in equations (1) to (4) and is based on the formulation we included previously [60] in the AP model formulated by ten Tusscher et al. [48].
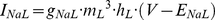
(1)

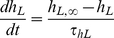
(2)

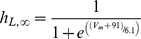
(3)

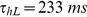
(4)We adopted these equations following Hodgkin Huxley formalism, where the activation gate (m_L_) and the Nernst potential for Na^+^ (E_NaL_) were unchanged with respect to I_NaT_ formulation in the GPB model. The steady-state inactivation gate (h_L∞_) was taken from Maltsev et al. [64], as did Hund et al. in their model for canine ventricular myocytes [46]. The maximum conductance (g*_NaL_*) and the time constant of inactivation (τ_hL_) were fitted to reproduce I*_NaL_* data taken from human mid-myocardial myocytes [58]. In their experiments, Maltsev et al. [58] measured an I*_NaL_*/I*_NaT_* ratio of approximately 0.1% using a voltage clamp protocol at room temperature. In our model g*_NaL_* was fitted accordingly using voltage clamp simulations, yielding 0.015 mS/µF. No correction factor for temperature was applied, as we assumed that I*_NaL_*/I*_NaT_* ratio does not change with temperature in myocytes, as reported in transfected HEK-293 cells [65]. However, because experimental data indicate that ion channel dynamics are altered by temperature, the time constant of inactivation (τ_hL_) of I_NaL_ reported by Maltsev et al. [26] for human ventricular mid-myocardial myocytes was multiplied by a Q_10_ factor of 2.2 [65], yielding 233 ms at 37°C.

### Heart failure cellular model for human

The remodeling of myocyte electrophysiology in HF is well described [3] and can explain for the most, the hallmark characteristics of failing cardiac tissues and myocytes, such as AP prolongation and alteration of Ca^2+^ and Na^+^ handling [2], [6], [66]. On the basis of experimental observations [3], [4], [7], [9], [11], [13]–[15], [67] and previous simulation studies [5], [12], [44], [47], we hereby propose various changes in the formulation of several ionic currents of the GPB model to reproduce the reported experimental changes in AP and intracellular Ca^2+^ and Na^+^ handling in ventricular myocytes from failing human hearts [8], [31], [66]. Our model will be referred to as the basic HF model. Table 1 summarizes the changes we made in the different ionic properties with respect to the GPB model. The ionic remodeling is mainly based on experimental data observed in human hearts.

**Table 1:** Heart failure remodeling.

	% Change vs. GPB	References	Experimental conditions
**I_NaL_**	↑ 200	Maltsev et al., 2007 [15]	Isolated cardiomyocytes from LV mid-myocardium of failing dog hearts. Whole cell voltage clamp (room temperature)
		Valdivia et al., 2005 [16]	Isolated cardiomyocytes from LV of failing human hearts. Whole cell voltage clamp (room temperature)
**ô_hL_**	↑ 200	Maltsev et al., 2007 [15]	Isolated cardiomyocytes from LV mid-myocardium of failing human hearts. Whole cell voltage clamp (room temperature)
**I_to_**	↓ 60	Wettwer et al., 1994 [70]	Isolated cardiomyocytes from LV endocardium of failing human hearts. Whole cell voltage clamp (room temperature)
		Beuckelmann et al., 1993 [10]	Isolated cardiomyocytes from LV mid-myocardium of failing human hearts. Whole cell voltage clamp (room temperature)
		Nabauer et al., 1996 [71]	Isolated cardiomyocytes from LV endocardium of failing human hearts. Whole cell voltage clamp (room temperature)
**I_K1_**	↓ 32	Tomaselli et al., 1999 [3]	Review article. Several species.
		Beuckelmann et al., 1993 [10]	Isolated cardiomyocytes from LV mid-myocardium of failing human hearts. Whole cell voltage clamp (35°C)
		Li et al., 2004 [7]	Isolated cardiomyocytes from RV epicardium of failing human hearts. Whole cell voltage clamp (room temperature)
**I_NaK_**	↓ 10	Bundgaard et al., 1996 [14]	Measurements of human myocardial. Na,K-ATPase concentration in failing hearts
		Tomaselli et al., 1999 [3]	Review article.
		Tomaselli et al. 2004 [4]	Review article.
**I_Nab_**	= 0	Priebe and Beuckelmann, 1998 [5]	Simulation of human HF.
**I_Cab_**	↑ 153	Priebe and Beuckelmann, 1998 [5]	Simulation of human HF.
**NCX**	↑ 175	Priebe and Beuckelmann, 1998 [5]	Simulation of human HF.
		Reinecke et al. 1996 [68]	The functional activity of the Na^+^-Ca^2+^ exchanger was determined by measuring the Na^+^-dependent Ca^2+^ uptake into membrane vesicles prepared from human left ventricular samples
**SERCA**	↓ 50	Piacentino et al., 2003 [11]	Isolated cardiomyocytes from LV of failing human hearts. Measurements of Ca^2+^ uptake rates by the SR (37°C).
		Hasenfuss et al., 1994 [72]	Endocardial strip preparations from human failing hearts. Measurements of Ca^2+^ uptake in myocardial homogenates (37°C).
		Schwinger et al., 1995 [73]	LV from human failing hearts. Measurements of Ca^2+^ uptake.
**I_leak_**	↑ 500	Bers et al., 2006 [9]	Review article.
**EC_50SR_**	↓ 11	Curran et al., 2010 [69]	Isolated cardiomyocytes from LV of failing rabbit hearts. Measurements of RyR sensitivity to SR Ca^2+^.
		Antoons et al., 2007 [13]	Review article.
		Bers et al., 2006 [9]	Review article.

Changes in ion channel, transporters, and pumps activities, and constants used in the basic heart failure (HF) model. The changes are indicated in percentage of increase (↑) or decrease (↓) with respect to the Grandi et al. model (the GPB model) [25].

A prominent increase in I_NaL_ and slowing of current decay has been described in ventricular mid-myocardial myocytes isolated from failing hearts of dogs and humans [15], [67]. Accordingly, the current density and the time constant of inactivation were increased two-fold compared to non-failing cells. Although Maltsev et al. [15] reported a smaller increase in both values in failing myocytes, their experiments were performed at room temperature and stated that the changes are expected to increase at physiological temperature. Furthermore, the values of the time constant of inactivation could be measured for human myocytes, but the current density was only given for dog myocytes, as the authors state the difficulty to measure it in human myocytes where the variability was very high. Also, in a previous study by Valdivia et al. [16], I_NaL_ was increased 5-fold in human failing myocytes with respect to normal myocytes. Downregulation of K^+^ currents is the most consistent ionic current change observed in myocytes isolated from failing hearts from animal models and humans [6], [7], [10]. Mainly, I_to_ is downregulated without a significant change in the voltage dependence or kinetics [10]. We reduced the transient outward K^+^ current (I_to_) to 40% of its normal value. Reported changes in I_K1_ functional expression are more variable than I_to_ [3], [10] and have a strong dependence on the etiology. The conductance of this ion channel was multiplied by 0.68 as in [12], a value within the experimental range. In regards to [Na^+^]_i_ handling, which is also altered in HF, we reduced I_NaK_ activity by 10%, as the preponderance of experimental data reveal that the expression and function of the Na^+^/K^+^-ATPase are reduced in HF [3], [4], [14]. Similar to that of Priebe and Beuckelmann simulation study [5], the background Na^2+^ current (I_Nab_) was not included in our HF model. Finally, the changes in intracellular and SR Ca^2+^ homeostasis were achieved by an increase of 75% of I_NCX_ [5], [12], [68], and a decrease of 50% of I_SERCA_ [11]. To reproduce the experimentally observed changes in Ca^2+^ sensitivity of the ryanodine receptor (RyR) [13], [69], I_leak_ was increased 3-fold and the parameter EC_50SR_ for SR [Ca^2+^]-dependent activation of SR release (see Grandi [25] supplementary data) was reduced to 0.4 mM. The background Ca^2+^ current was changed as in the study of Priebe and Beuckelmann [5].

### Sensitivity analysis of the HF model

A sensitivity analysis was performed to investigate how the reported variability in HF remodeling data might modulate the main EP characteristics in HF. These characteristics “c” include APD at 90% and 50% of repolarization (APD_90_ and APD_50_), triangulation, peak systolic and diastolic [Ca^2+^]_i_ transient, ô_Ca_ decay, peak systolic and diastolic [Ca^2+^]_SR_, [Na^+^]_i_, and t_NCXRP_. These characteristics were calculated at steady-state HF conditions (HF basic model) and after varying one parameter “p” at a time. The parameters included each of the ionic current properties modified in the HF basic model, and were varied to its normal value, as in the GPB model, and to a value representing twice that observed in the HF basic remodeling. Although an important change has been implemented (double change) to be considered as a high variability, only the sensitivity, i.e. the relative effect was analyzed. The sensitivity analysis performed similar to that of Romero et al. [32], wherein the indexes percentage of change (D_c,p,x_) and sensitivities (S_c,p_) were calculated as follows:
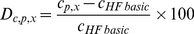
(5)

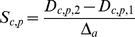
(6)with C_p,x_ and C_HFbasic_ being the magnitude of the characteristic c when parameter p undergoes a double change (x = 2) with respect to the HF basic change, or no change at all (x = 1); Δ_a_ is the total interval of change of parameter p.

### Stimulation protocols

Voltage clamp was used to simulate I_NaL_ behavior. A voltage pulse to −20 mV was applied from a holding potential of −120 mV as depicted in the inset of Figure 1A. To calculate I_NaL_/I_NaT_ ratios (see Figure 1C) I_NaL_ was measured at 40 ms after the application of the depolarizing pulse and was divided by peak I_NaT._

Cellular simulations were conducted at a stimulation rate of 1 Hz. Measurements were taken after achieving steady-state conditions.

Changes in [Na^+^]_i_ and [Ca^2+^]_i_ levels at various stimulation rates were measured using a staircase protocol as previously described [32], [33]. Cardiomyocytes were stimulated at increasing fast frequencies (0.5, 1, 1.5, 2 and 2.5 Hz) for 10 minutes, and [Ca^2+^]_i_ and [Na^+^]_i_ levels were recorded for each of the frequencies.

To analyze the APD rate-dependence, simulations were performed at different frequencies of stimulation (0.5, 1, 1.25 and 1.6 Hz). Measurements of APD_90_ were taken after achieving the steady-state; likewise for APs, [Na^+^]_i_ and several ionic currents. The rate-dependence was measured as the difference between the maximum APD_90_ for a stimulation rate of 0.5 Hz and the minimum APD_90_ corresponding to the highest stimulation frequency.

## Supporting Information

Figure S1 **Sensitivity of APD_90_ to the I_NaL_ amplitude in HF.** Steady-state APs at 1 Hz (square symbols and solid lines) and 0.5 Hz (circle symbols and dashed lines) pacing rates with varying I_NaL_/I_NaT_ for normal conditions using the GPB model (thick lines) and under basic HF conditions (thin lines) where I_NaL_ is doubled with respect to normal conditions. The range of experimental APD_90_ for human for normal conditions is represented by the two dotted blue lines. The range of experimental APD_90_ for human for HF conditions is represented by the two discontinuous red lines.
(TIF)

Figure S2 **Sensitivity of APD rate-dependence to variations in individual ionic parameters in HF.** The steady-state APD_90_ for different stimulation frequencies is shown for normal conditions using the GPB model (thick line), for basic HF conditions (solid line), and for a 15% increase (long dashed line) and a 15% reduction (short dashed line) of one ionic parameter with respect to its value in the basic HF model.
(TIF)

Figure S3 **Sensitivity of APD rate-dependence to variations in all ionic parameters in HF.** The steady-state APD_90_ for different stimulation frequencies is shown for normal conditions using the GPB model (thick line), for basic HF conditions (solid line), and for a 15% increase (long dashed line) and a 15% reduction (short dashed line) of all the ionic parameters simultaneously with respect to their value in the basic HF model.
(TIF)

Figure S4 **Sensitivity of rate-dependent changes in systolic [Ca^2+^]_i_ to variations in individual ionic parameters in HF.** Systolic [Ca^2+^]_i_ after 10 minutes of stimulation at increasing rates is shown for normal conditions using the GPB model (thick line), for basic HF conditions (solid line), and for a 15% increase (long dashed line) and a 15% reduction (short dashed line) of one ionic parameter with respect to its value in the basic HF model.
(TIF)

Figure S5 **Sensitivity of rate-dependent changes in systolic [Ca^2+^]_i_ to variations in all ionic parameters in HF.** Systolic [Ca^2+^]_i_ after 10 minutes of stimulation at increasing rates is shown for normal conditions using the GPB model (thick line), for basic HF conditions (solid line), and for a 15% increase (long dashed line) and a 15% reduction (short dashed line) of all the ionic parameters simultaneously with respect to their value in the basic HF model.
(TIF)

Figure S6 **Sensitivity of rate-dependent changes in diastolic [Ca^2+^]_i_ to variations in individual ionic parameters in HF.** Diastolic [Ca^2+^]_i_ after 10 minutes of stimulation at increasing rates is shown for normal conditions using the GPB model (thick line), for basic HF conditions (solid line), and for a 15% increase (long dashed line) and a 15% reduction (short dashed line) of one ionic parameter with respect to its value in the basic HF model.
(TIF)

Figure S7 **Sensitivity of rate-dependent changes in diastolic [Ca^2+^]_i_ to variations in all ionic parameters in HF.** Diastolic [Ca^2+^]_i_ after 10 minutes of stimulation at increasing rates is shown for normal conditions using the GPB model (thick line), for basic HF conditions (solid line), and for a 15% increase (long dashed line) and a 15% reduction (short dashed line) of all the ionic parameters simultaneously with respect to their value in the basic HF model.
(TIF)

Figure S8 **Sensitivity of rate-dependent changes in [Na^+^]_i_ to variations in individual ionic parameters in HF.** [Na^+^]_i_ after 10 minutes of stimulation at increasing rates is shown for normal conditions using the GPB model (thick line), for basic HF conditions (solid line), and for a 15% increase (long dashed line) and a 15% reduction (short dashed line) of one ionic parameter with respect to its value in the basic HF model.
(TIF)

Figure S9 **Sensitivity of rate-dependent changes in [Na^+^]_i_ to variations in all ionic parameters in HF.** [Na^+^]_i_ after 10 minutes of stimulation at increasing rates is shown for normal conditions using the GPB model (thick line), for basic HF conditions (solid line), and for a 15% increase (long dashed line) and a 15% reduction (short dashed line) of all the ionic parameters simultaneously with respect to their value in the basic HF model.
(TIF)

Figure S10 **Sensitivity of EAD generation to variations in individual ionic parameters in HF.** Steady-state APs at 1-Hz pacing rate with 50% inhibition of I_Kr_, 30% increase of I_CaL_. The simulated results using the basic HF model are shown with a thick line, the solid and dashed lines show the results obtained for a 15% increase and a 15% reduction, respectively, of one ionic parameter with respect to its value in the basic HF model.
(TIF)

Figure S11 **Sensitivity of EAD generation to variations in all ionic parameters in HF.** Steady-state APs at 1-Hz pacing rate with 50% inhibition of I_Kr_, 30% increase of I_CaL_. The simulated results using the basic HF model are shown with a thick line, the solid and dashed lines show the results obtained for a 15% increase and a 15% reduction, respectively, of all the ionic parameters simultaneously with respect to their value in the basic HF model.
(TIF)

Figure S12 **Mechanisms for early afterdepolarizations with a 50% continuous block of I_rel_.** Simulated APs and ionic currents at 1-Hz pacing rate under HF conditions, 50% inhibition of I_Kr_, 30% increase of I_CaL_. Panel A shows EADs (dark line) with the basic HF value of I_rel_ and APs with no EADs when I_rel_ was 50% blocked (light line). The temporal evolutions of I_NaL_ (panel B), [Ca^2+^]_SR_ (panel C), I_rel_ (panel D), NCX activity (panel E), and activation gate of I_CaL_ (panel F) are also depicted with the basic HF value of I_rel_ (dark line) and when I_rel_ was 50% blocked (light line).
(TIF)

Figure S13 **Mechanisms for early afterdepolarizations with a 50% transitory block of I_rel_.** Simulated APs and ionic currents at 1-Hz pacing rate under HF conditions, 50% inhibition of I_Kr_, 30% increase of I_CaL_. Panel A shows EADs (dark line) with the basic HF value of I_rel_ and APs with no EADs when I_rel_ was 50% blocked (light line) during the 5 stimulations indicated by a horizontal line. The temporal evolutions of I_NaL_ (panel B), [Ca^2+^]_SR_ (panel C), I_rel_ (panel D), NCX activity (panel E), and activation gate of I_CaL_ (panel F) are also depicted with the basic HF value of I_rel_ (dark line) and when I_rel_ was 50% blocked (light line) during the 5 stimulations indicated by a horizontal line.
(TIF)

Figure S14 **Mechanisms for early afterdepolarizations with a 50% continuous block of I_NCX_.** Simulated APs and ionic currents at 1-Hz pacing rate under HF conditions, 50% inhibition of I_Kr_, 30% increase of I_CaL_. Panel A shows EADs (dark line) with the basic HF value of I_NCX_ and APs with no EADs when I_NCX_ was 50% blocked (light line). The temporal evolutions of I_NaL_ (panel B), [Ca^2+^]_SR_ (panel C), I_rel_ (panel D), NCX activity (panel E), and activation gate of I_CaL_ (panel F) are also depicted with the basic HF value of I_NCX_ (dark line) and when I_NCX_ was 50% blocked (light line).
(TIF)

Figure S15 **Mechanisms for early afterdepolarizations with a 50% transitory block of I_NCX_.** Simulated APs and ionic currents at 1-Hz pacing rate under HF conditions, 50% inhibition of I_Kr_, 30% increase of I_CaL_. Panel A shows EADs (dark line) with the basic HF value of I_NCX_ and APs with no EADs when I_NCX_ was 50% blocked (light line during the 5 stimulations indicated by a horizontal line. The temporal evolutions of I_NaL_ (panel B), [Ca^2+^]_SR_ (panel C), I_rel_ (panel D), NCX activity (panel E), and activation gate of I_CaL_ (panel F) are also depicted with the basic HF value of I_NCX_ (dark line) and when I_NCX_ was 50% blocked (light line) during the 5 stimulations indicated by a horizontal line.
(TIF)
