# Supplementary figures and images for: Simulation and Mechanistic Investigation of the Arrhythmogenic Role of the Late Sodium Current in Human Heart Failure
Source: PLoS One. 2012 Mar 12;7(3):e32659. doi: 10.1371/journal.pone.0032659 (PMC3299678; doi:10.1371/journal.pone.0032659)

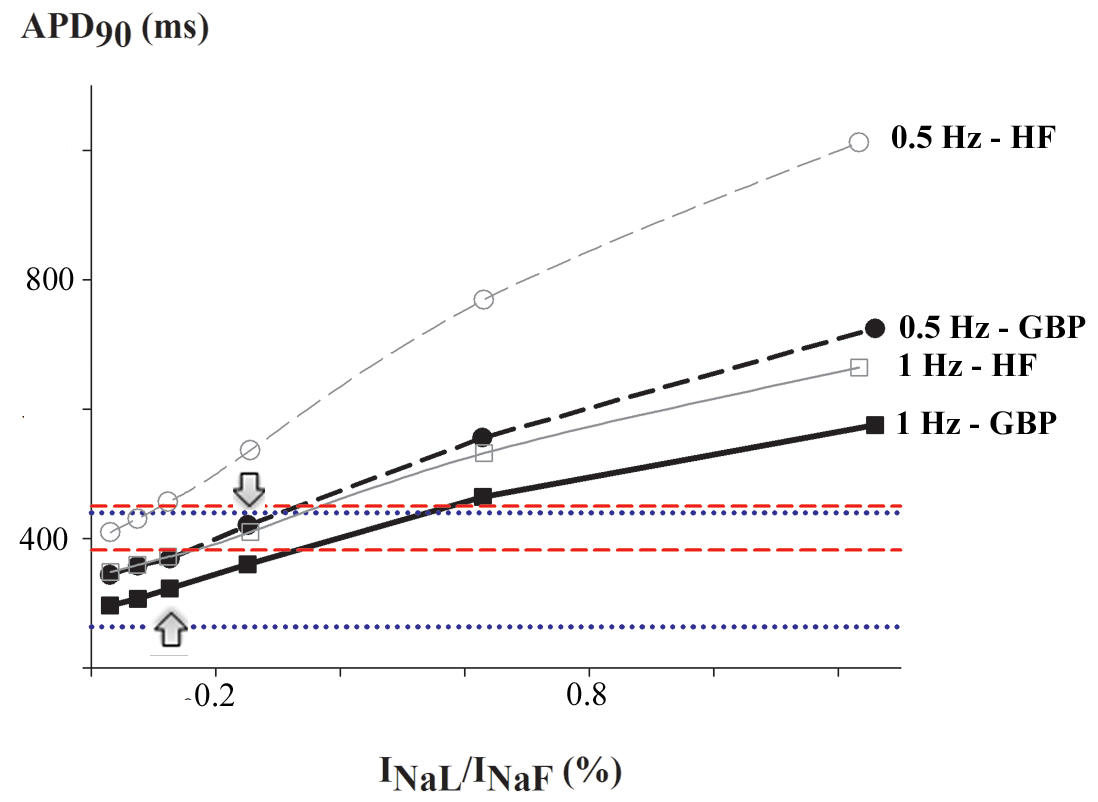

Supplement: Figure S1 — Sensitivity of APD90 to the INaL amplitude in HF. Steady-state APs at 1 Hz (square symbols and solid lines) and 0.5 Hz (circle symbols and dashed lines) pacing rates with varying INaL/INaT for normal conditions using the GPB model (thick lines) and under basic HF conditions (thin lines) where INaL is doubled with respect to normal conditions. The range of experimental APD90 for human for normal conditions is represented by the two dotted blue lines. The range of experimental APD90 for human for HF conditions is represented by the two discontinuous red lines. (TIF) [file pone.0032659.s001.tif]

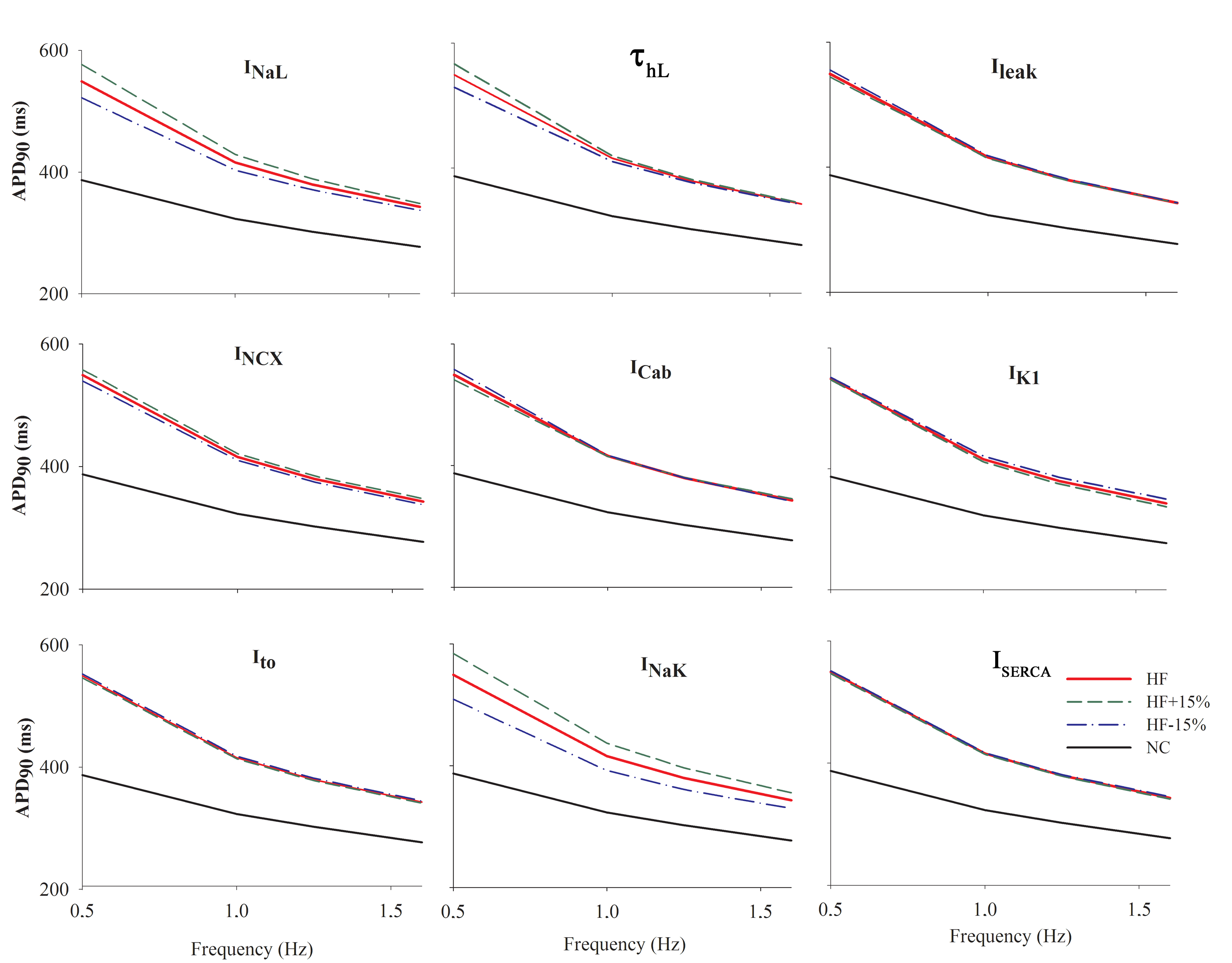

Supplement: Figure S2 — Sensitivity of APD rate-dependence to variations in individual ionic parameters in HF. The steady-state APD90 for different stimulation frequencies is shown for normal conditions using the GPB model (thick line), for basic HF conditions (solid line), and for a 15% increase (long dashed line) and a 15% reduction (short dashed line) of one ionic parameter with respect to its value in the basic HF model. (TIF) [file pone.0032659.s002.tif]

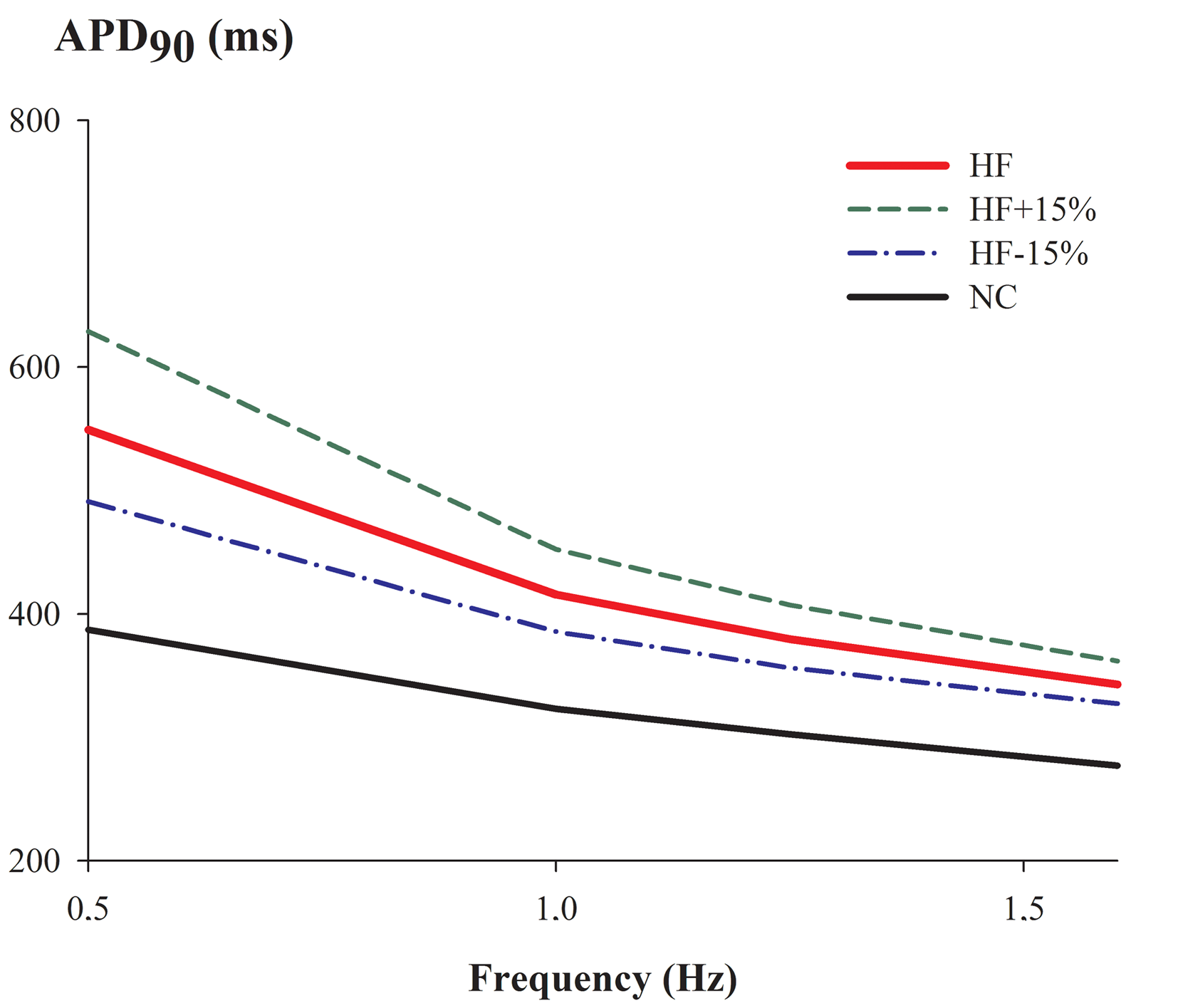

Supplement: Figure S3 — Sensitivity of APD rate-dependence to variations in all ionic parameters in HF. The steady-state APD90 for different stimulation frequencies is shown for normal conditions using the GPB model (thick line), for basic HF conditions (solid line), and for a 15% increase (long dashed line) and a 15% reduction (short dashed line) of all the ionic parameters simultaneously with respect to their value in the basic HF model. (TIF) [file pone.0032659.s003.tif]

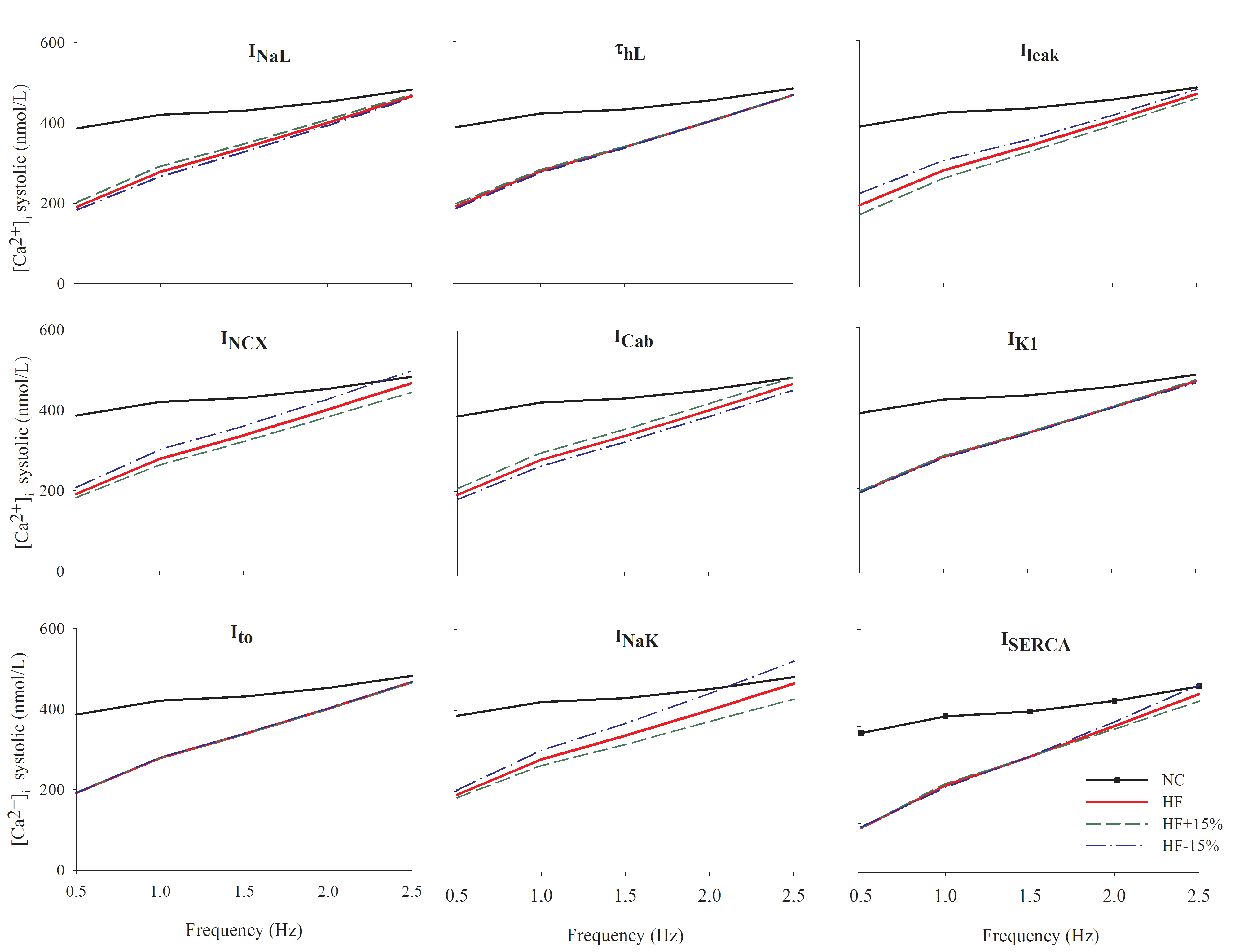

Supplement: Figure S4 — Sensitivity of rate-dependent changes in systolic [Ca2+]i to variations in individual ionic parameters in HF. Systolic [Ca2+]i after 10 minutes of stimulation at increasing rates is shown for normal conditions using the GPB model (thick line), for basic HF conditions (solid line), and for a 15% increase (long dashed line) and a 15% reduction (short dashed line) of one ionic parameter with respect to its value in the basic HF model. (TIF) [file pone.0032659.s004.tif]

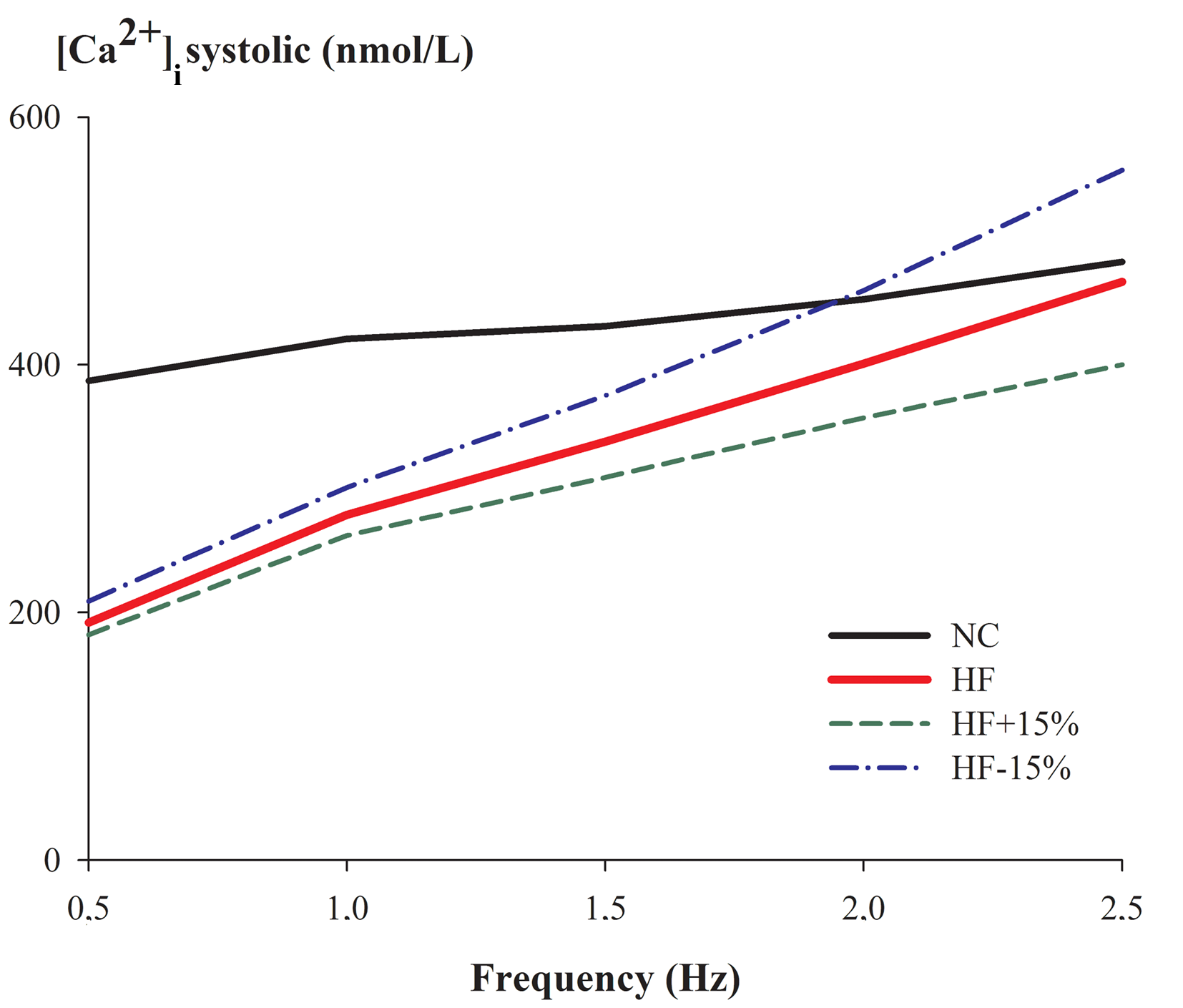

Supplement: Figure S5 — Sensitivity of rate-dependent changes in systolic [Ca2+]i to variations in all ionic parameters in HF. Systolic [Ca2+]i after 10 minutes of stimulation at increasing rates is shown for normal conditions using the GPB model (thick line), for basic HF conditions (solid line), and for a 15% increase (long dashed line) and a 15% reduction (short dashed line) of all the ionic parameters simultaneously with respect to their value in the basic HF model. (TIF) [file pone.0032659.s005.tif]

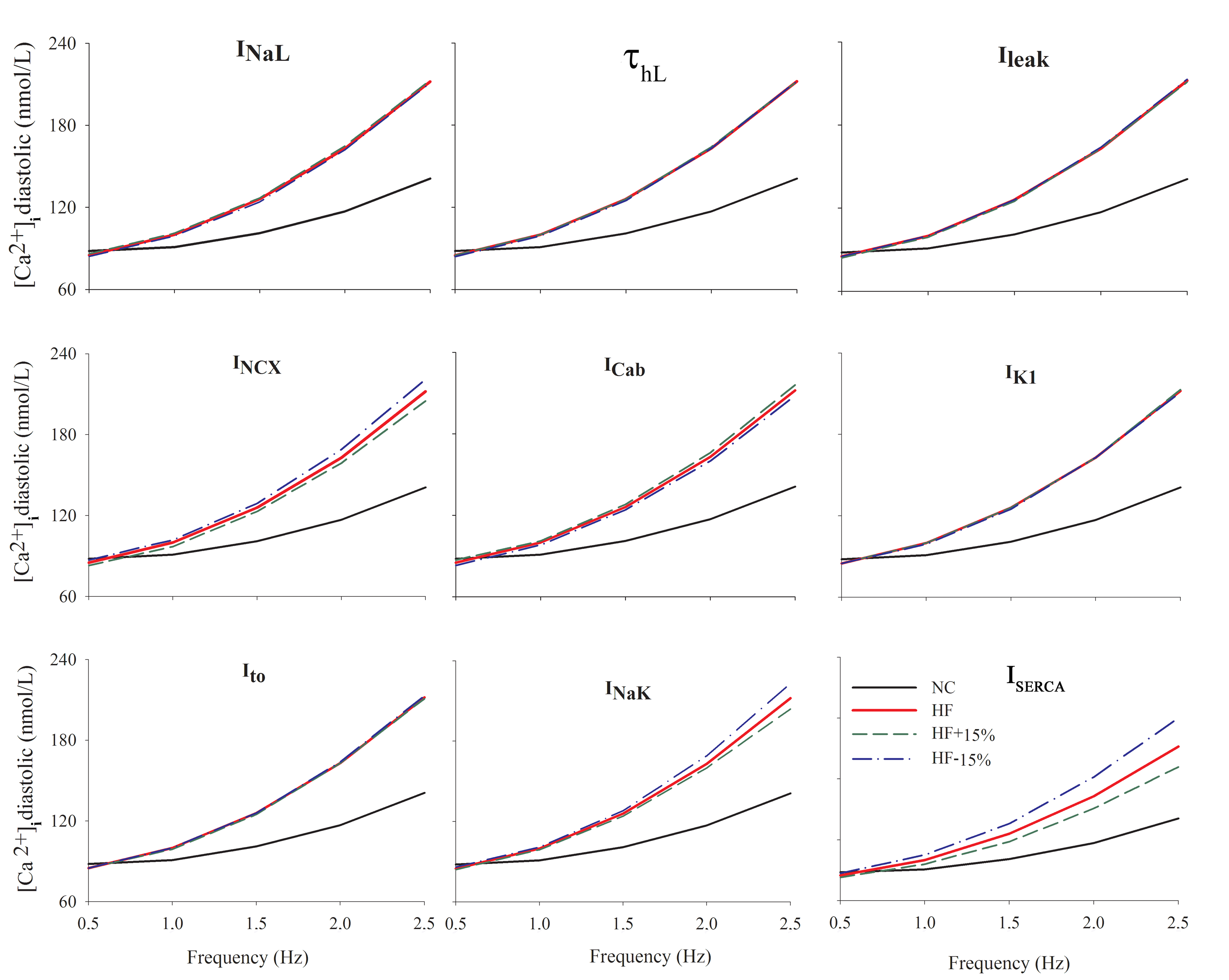

Supplement: Figure S6 — Sensitivity of rate-dependent changes in diastolic [Ca2+]i to variations in individual ionic parameters in HF. Diastolic [Ca2+]i after 10 minutes of stimulation at increasing rates is shown for normal conditions using the GPB model (thick line), for basic HF conditions (solid line), and for a 15% increase (long dashed line) and a 15% reduction (short dashed line) of one ionic parameter with respect to its value in the basic HF model. (TIF) [file pone.0032659.s006.tif]

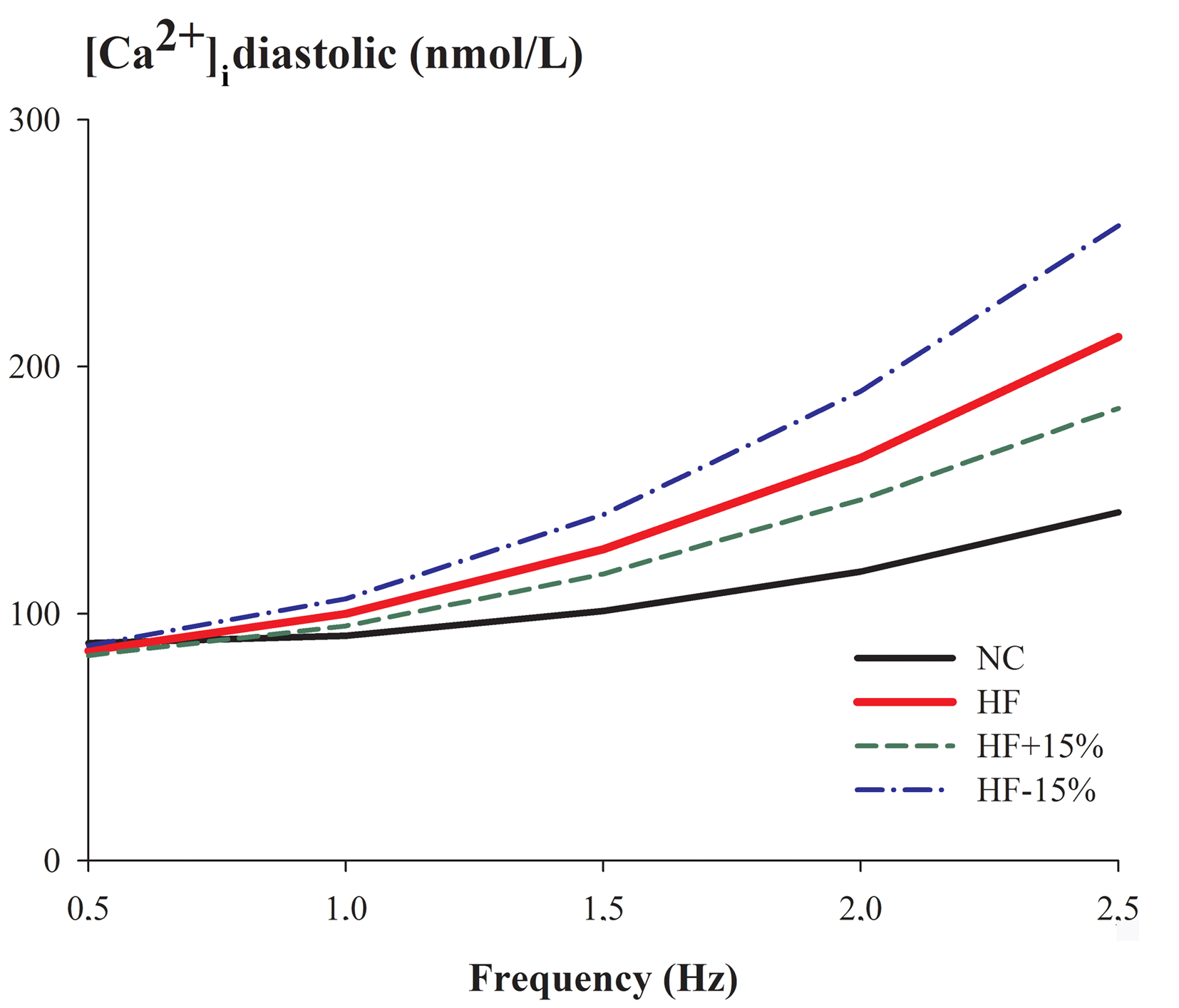

Supplement: Figure S7 — Sensitivity of rate-dependent changes in diastolic [Ca2+]i to variations in all ionic parameters in HF. Diastolic [Ca2+]i after 10 minutes of stimulation at increasing rates is shown for normal conditions using the GPB model (thick line), for basic HF conditions (solid line), and for a 15% increase (long dashed line) and a 15% reduction (short dashed line) of all the ionic parameters simultaneously with respect to their value in the basic HF model. (TIF) [file pone.0032659.s007.tif]

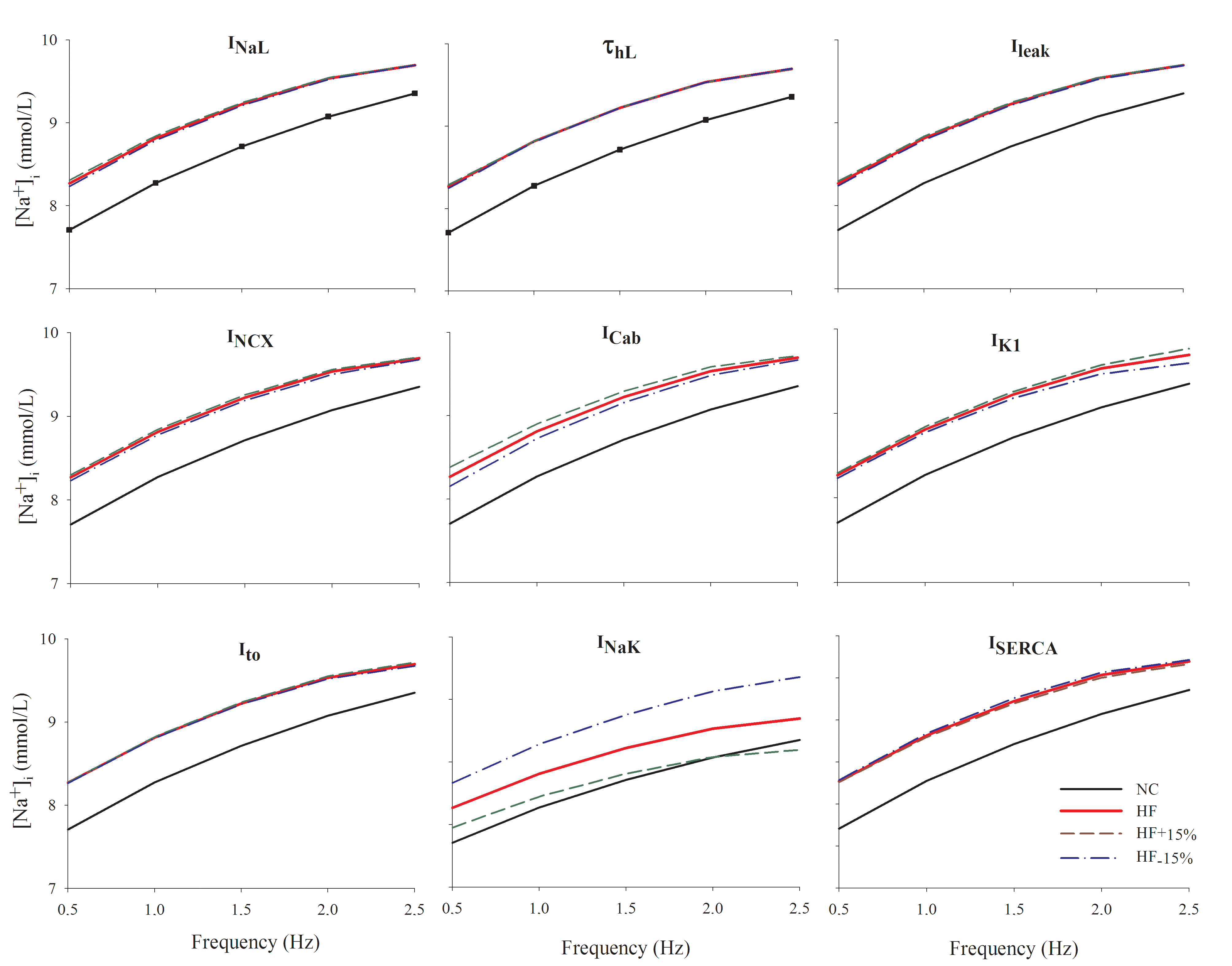

Supplement: Figure S8 — Sensitivity of rate-dependent changes in [Na+]i to variations in individual ionic parameters in HF. [Na+]i after 10 minutes of stimulation at increasing rates is shown for normal conditions using the GPB model (thick line), for basic HF conditions (solid line), and for a 15% increase (long dashed line) and a 15% reduction (short dashed line) of one ionic parameter with respect to its value in the basic HF model. (TIF) [file pone.0032659.s008.tif]

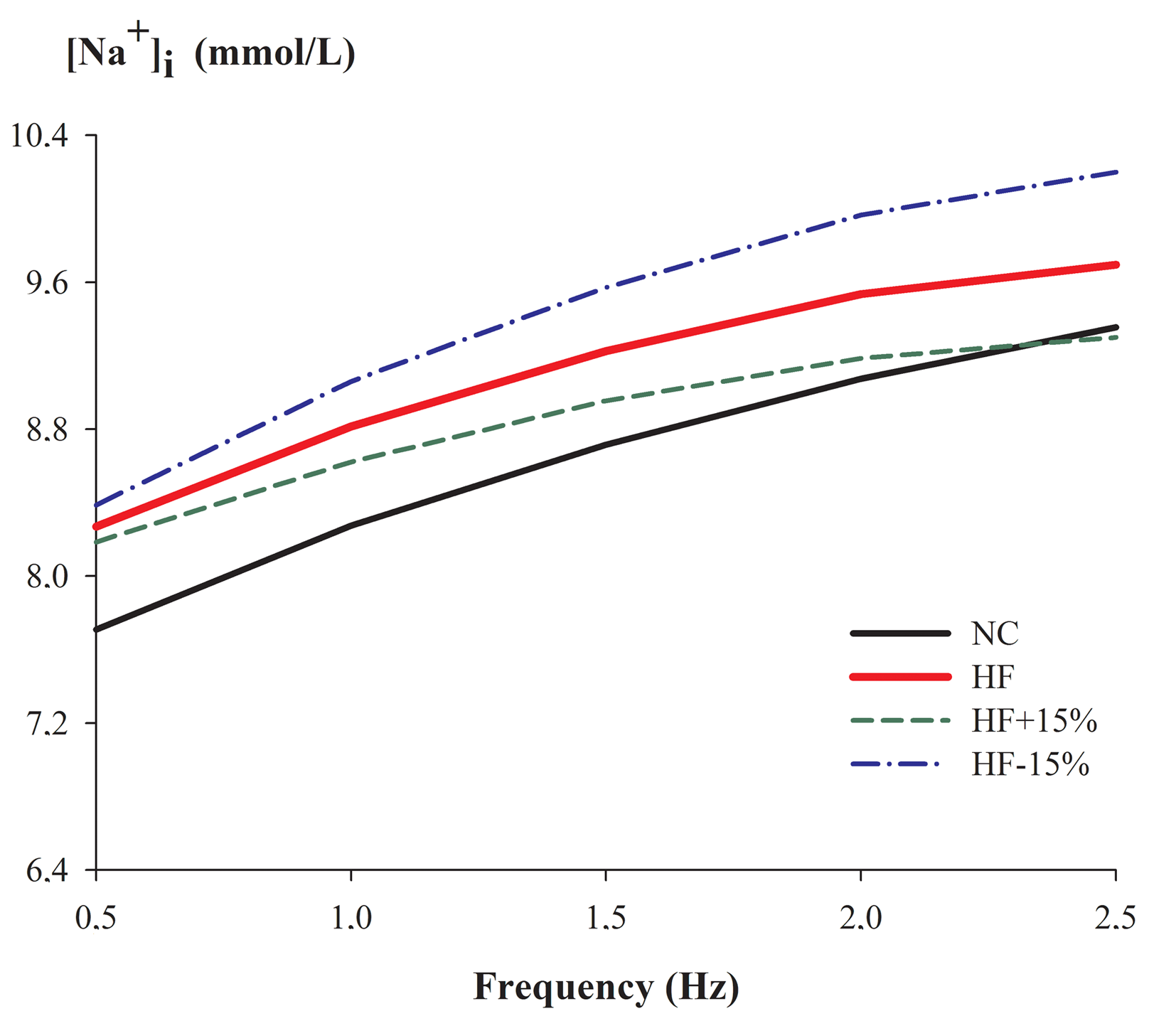

Supplement: Figure S9 — Sensitivity of rate-dependent changes in [Na+]i to variations in all ionic parameters in HF. [Na+]i after 10 minutes of stimulation at increasing rates is shown for normal conditions using the GPB model (thick line), for basic HF conditions (solid line), and for a 15% increase (long dashed line) and a 15% reduction (short dashed line) of all the ionic parameters simultaneously with respect to their value in the basic HF model. (TIF) [file pone.0032659.s009.tif]

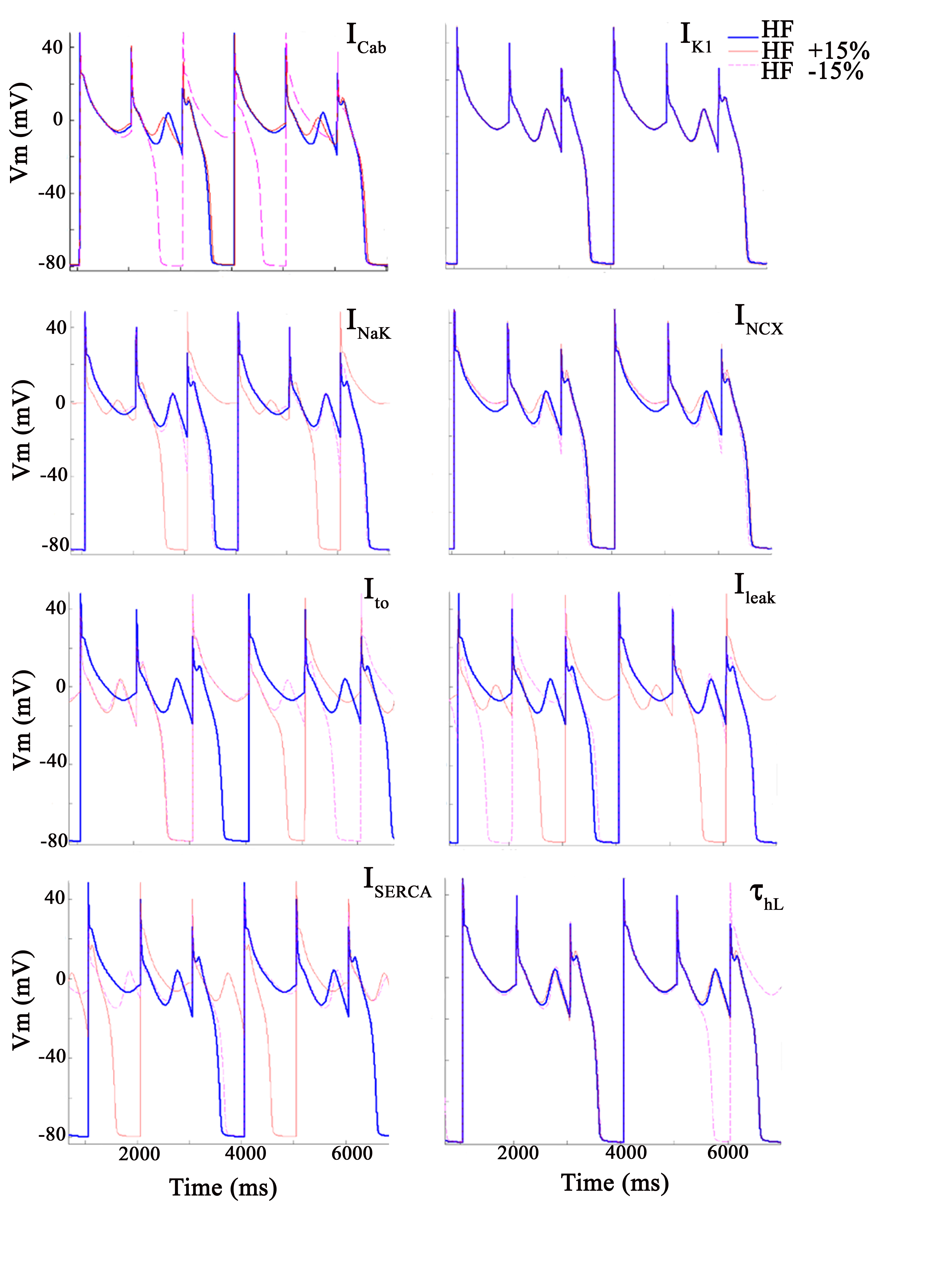

Supplement: Figure S10 — Sensitivity of EAD generation to variations in individual ionic parameters in HF. Steady-state APs at 1-Hz pacing rate with 50% inhibition of IKr, 30% increase of ICaL. The simulated results using the basic HF model are shown with a thick line, the solid and dashed lines show the results obtained for a 15% increase and a 15% reduction, respectively, of one ionic parameter with respect to its value in the basic HF model. (TIF) [file pone.0032659.s010.tif]

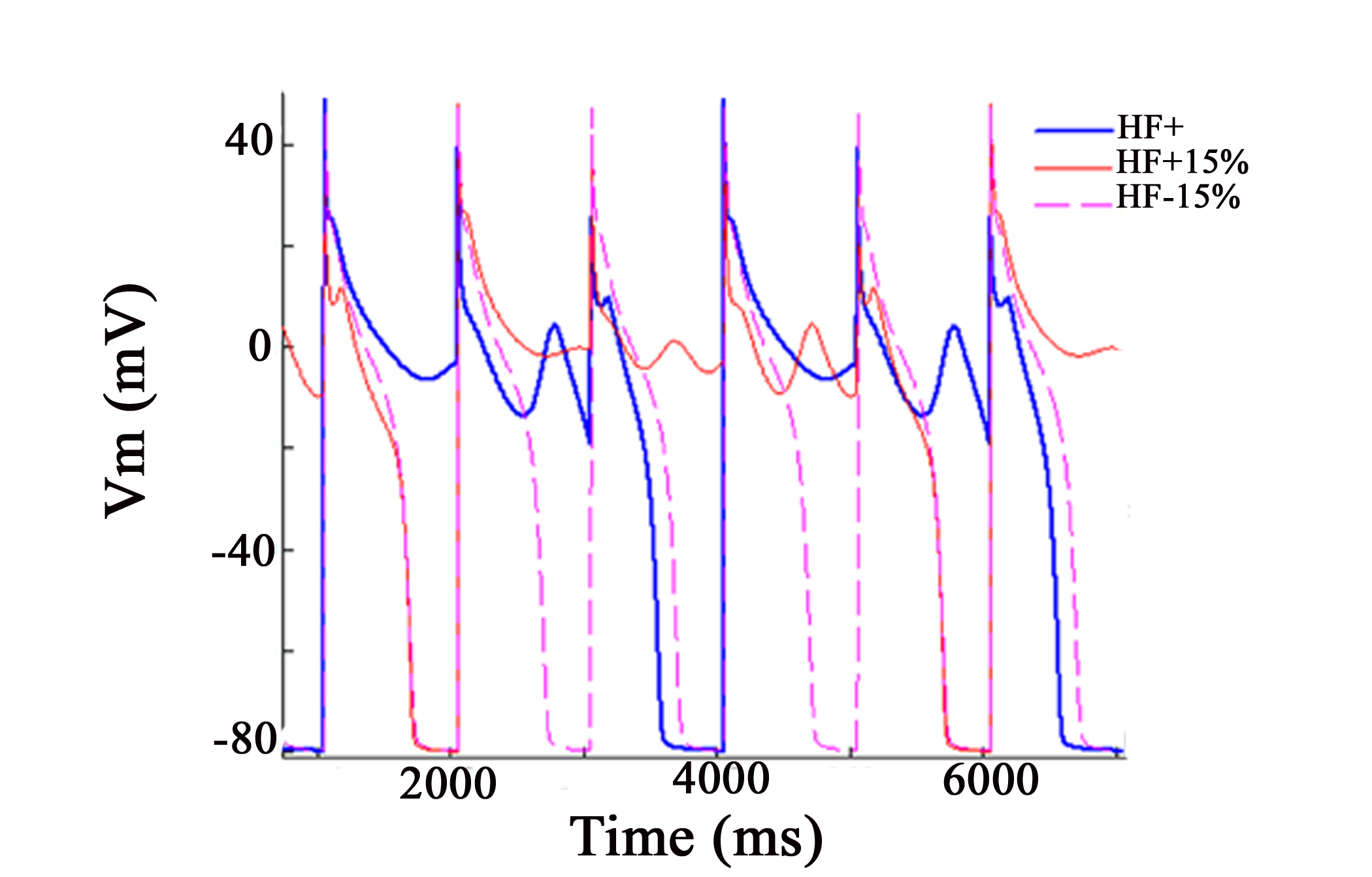

Supplement: Figure S11 — Sensitivity of EAD generation to variations in all ionic parameters in HF. Steady-state APs at 1-Hz pacing rate with 50% inhibition of IKr, 30% increase of ICaL. The simulated results using the basic HF model are shown with a thick line, the solid and dashed lines show the results obtained for a 15% increase and a 15% reduction, respectively, of all the ionic parameters simultaneously with respect to their value in the basic HF model. (TIF) [file pone.0032659.s011.tif]

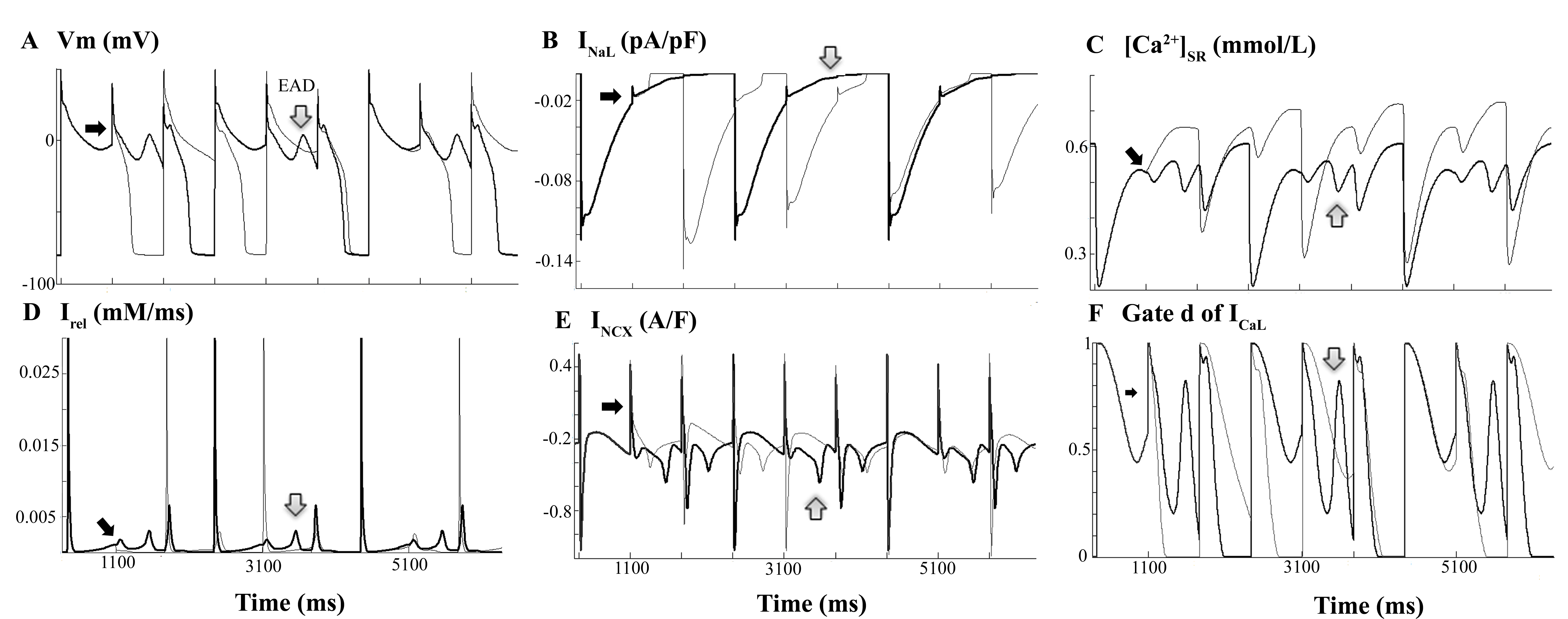

Supplement: Figure S12 — Mechanisms for early afterdepolarizations with a 50% continuous block of Irel. Simulated APs and ionic currents at 1-Hz pacing rate under HF conditions, 50% inhibition of IKr, 30% increase of ICaL. Panel A shows EADs (dark line) with the basic HF value of Irel and APs with no EADs when Irel was 50% blocked (light line). The temporal evolutions of INaL (panel B), [Ca2+]SR (panel C), Irel (panel D), NCX activity (panel E), and activation gate of ICaL (panel F) are also depicted with the basic HF value of Irel (dark line) and when Irel was 50% blocked (light line). (TIF) [file pone.0032659.s012.tif]

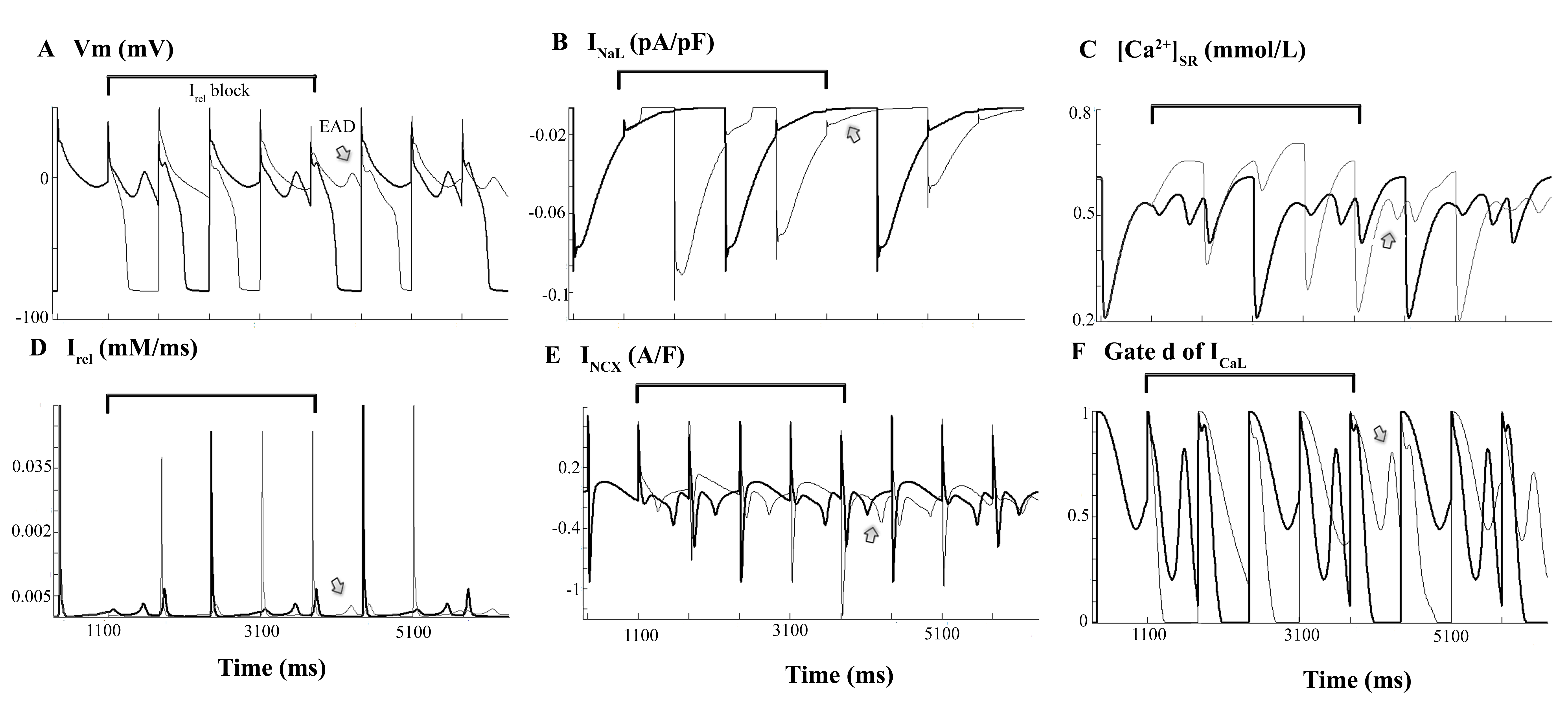

Supplement: Figure S13 — Mechanisms for early afterdepolarizations with a 50% transitory block of Irel. Simulated APs and ionic currents at 1-Hz pacing rate under HF conditions, 50% inhibition of IKr, 30% increase of ICaL. Panel A shows EADs (dark line) with the basic HF value of Irel and APs with no EADs when Irel was 50% blocked (light line) during the 5 stimulations indicated by a horizontal line. The temporal evolutions of INaL (panel B), [Ca2+]SR (panel C), Irel (panel D), NCX activity (panel E), and activation gate of ICaL (panel F) are also depicted with the basic HF value of Irel (dark line) and when Irel was 50% blocked (light line) during the 5 stimulations indicated by a horizontal line. (TIF) [file pone.0032659.s013.tif]

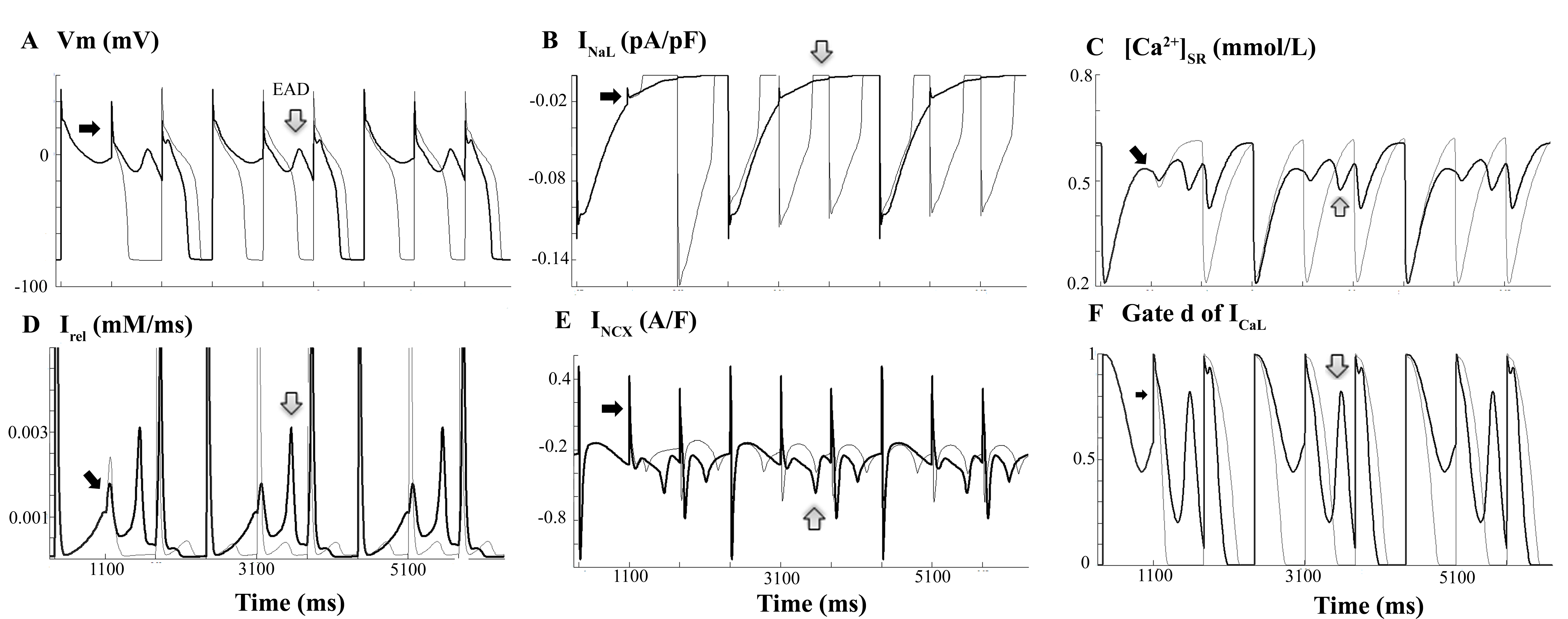

Supplement: Figure S14 — Mechanisms for early afterdepolarizations with a 50% continuous block of INCX. Simulated APs and ionic currents at 1-Hz pacing rate under HF conditions, 50% inhibition of IKr, 30% increase of ICaL. Panel A shows EADs (dark line) with the basic HF value of INCX and APs with no EADs when INCX was 50% blocked (light line). The temporal evolutions of INaL (panel B), [Ca2+]SR (panel C), Irel (panel D), NCX activity (panel E), and activation gate of ICaL (panel F) are also depicted with the basic HF value of INCX (dark line) and when INCX was 50% blocked (light line). (TIF) [file pone.0032659.s014.tif]

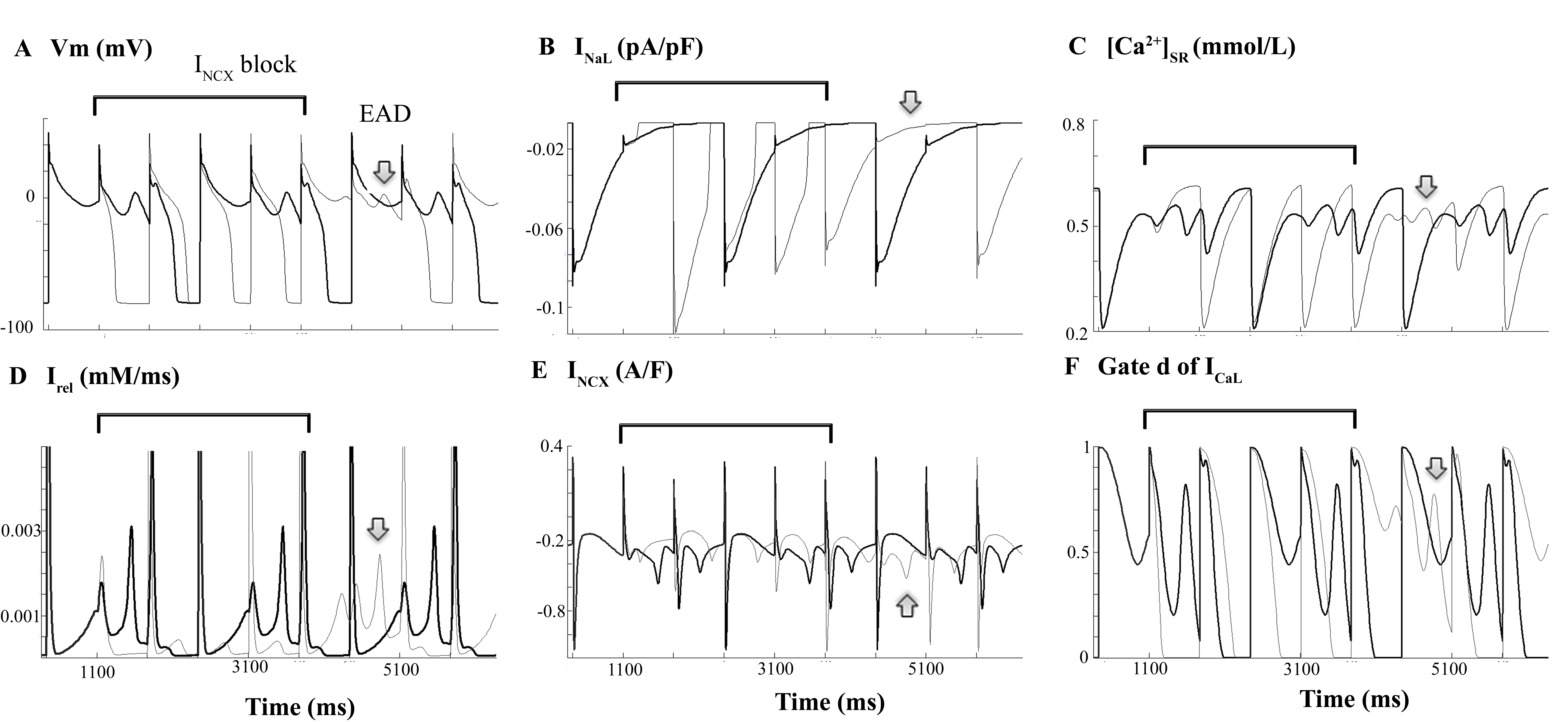

Supplement: Figure S15 — Mechanisms for early afterdepolarizations with a 50% transitory block of INCX. Simulated APs and ionic currents at 1-Hz pacing rate under HF conditions, 50% inhibition of IKr, 30% increase of ICaL. Panel A shows EADs (dark line) with the basic HF value of INCX and APs with no EADs when INCX was 50% blocked (light line during the 5 stimulations indicated by a horizontal line. The temporal evolutions of INaL (panel B), [Ca2+]SR (panel C), Irel (panel D), NCX activity (panel E), and activation gate of ICaL (panel F) are also depicted with the basic HF value of INCX (dark line) and when INCX was 50% blocked (light line) during the 5 stimulations indicated by a horizontal line. (TIF) [file pone.0032659.s015.tif]
